# Developmental and tissue-specific expression of thyrotropin-releasing hormone signaling genes in zebrafish and its association with glycemic regulation

**DOI:** 10.1242/bio.062418

**Published:** 2026-04-02

**Authors:** David Díaz-Ortegón, Aurea Orozco, Santiago M. Pech-Pool, María Carlota García-Gutierrez, Iván Lazcano

**Affiliations:** ^1^ Laboratorio de Epidemiología Traslacional, Centro de Investigación Biomédica Avanzada, Facultad de Medicina, Universidad Autónoma de Querétaro, Querétaro 76010, Mexico; ^2^ Instituto de Neurobiología, Universidad Nacional Autónoma de México, Campus Juriquilla, Querétaro 76230, Mexico; ^3^ Escuela Nacional de Estudios Superiores, Unidad Juriquilla, Universidad Nacional Autónoma de México, Querétaro 76230, Mexico

**Keywords:** TRH, TRH-R, TRH-DE, Glycemic metabolism, Zebrafish

## Abstract

Thyrotropin-releasing hormone (TRH) is a highly conserved tripeptide classically associated with regulation of the hypothalamic–pituitary–thyroid axis; however, TRH and its receptors are also widely distributed in peripheral metabolic tissues. While TRH has been implicated in glucose homeostasis in mammals, the developmental expression, tissue distribution, and sex-dependent metabolic roles of the TRHergic system in zebrafish remain poorly defined. Here, we characterized the TRHergic system in zebrafish (*Danio rerio*) by integrating bioinformatic analyses with developmental and adult expression profiling in both sexes, together with functional assessment under hyperglycemic conditions. TRHergic components – including pre proTRH, TRH receptor isoforms, and the TRH-degrading enzyme – were structurally conserved and dynamically expressed during early development, with *trh-r1b* displaying the most pronounced developmental changes. In adults, these genes exhibited broad central and peripheral expression with marked sex-specific patterns. *In vivo*, exogenous TRH reduced blood glucose levels in hyperglycemic males but not females. This effect was not associated with a direct insulinotropic response, supporting a modulatory role for TRH in metabolic regulation, likely involving peripheral tissues such as the pancreas and liver. Together, these findings establish zebrafish as a valuable vertebrate model for studying sex-dependent metabolic functions of the TRHergic system, particularly in the context of sex-dependent regulation of glucose homeostasis.

## Teaser

**Summary:** This study integratively characterizes the zebrafish TRHergic system, demonstrating early developmental activation, tissue- and sex-specific expression, and a conserved metabolic role in glycemic regulation.

## INTRODUCTION

Thyrotropin-releasing hormone (TRH) is an endocrine messenger initially known for its role in stimulating the synthesis and release of thyroid-stimulating hormone (TSH) from the anterior pituitary of mammals (Bøler et al., 1969; Burgus et al., 1969), and, later, as a key component of the hypothalamic-pituitary-thyroid (HPT) axis (reviewed in: Costa-E-Sousa et al., 2023). However, it is now known that this hormone is also expressed in extra-hypothalamic regions of the central nervous system (CNS), as well as in various peripheral tissues (reviewed in Fröhlich and Wahl, 2019). In vertebrates, TRH exerts its effects through its receptors (TRH-Rs), which belong to the G protein-coupled receptor (GPCR) superfamily and are widely distributed throughout the CNS and peripheral tissues (Heuer et al., 2000; Li et al., 2020; Lu et al., 2003; O'Dowd et al., 2000; Sun et al., 2003). After docking with TRH, these receptors trigger intracellular signaling cascades involving phospholipase activation and intracellular calcium mobilization, thereby eliciting TRH's diverse biological effects, such as thermoregulation, metabolic control, and endocrine hormone secretion (Sun et al., 2003; Trubacova et al., 2022). On the other hand, TRH degradation is primarily mediated by the thyrotropin-releasing hormone-degrading ectoenzyme (TRH-DE), which regulates its extracellular levels and biological activity (Charli et al., 2020; Lazcano et al., 2012, 2015).

In addition to its classical hypophysiotropic role in regulating adenohypophysis hormone secretion, TRH is now recognized as a pleiotropic peptide involved in diverse physiological processes, including the modulation of energy metabolism and blood glucose regulation (Morley et al., 1977; Bacová et al., 2006; Lechan and Fekete, 2006; Pekary et al., 2006; Štrbák, 2018). In this context, TRH expression has been identified in pancreatic β cells (Basmaciogullari et al., 2000; Mulla et al., 2009), and several studies have demonstrated that TRH can influence pancreatic hormone secretion and peripheral glucose utilization (Amir, 1988; Luo and Yano, 2004; Luo et al., 2014; Yamada et al., 2003). For instance, TRH-deficient mice develop fasting hyperglycemia (Yamada et al., 1997), and exogenous TRH modulates insulin release (Luo et al., 2008), suggesting the influence of this peptide in glycemic homeostasis.

Despite the high evolutionary conservation of TRH and its signaling components (TRH-Rs and TRH-DE), the hypophysiotropic role of TRH varies across vertebrate lineages (Galas et al., 2009; Lazcano et al., 2025). In several non-mammalian species, particularly teleost fish, regulation of the HPT axis is not exclusively dependent on TRH (Bernier et al., 2009; Lazcano et al., 2021). Instead, other neuropeptides, such as corticotropin-releasing hormone or gonadotropin-releasing hormone, can stimulate TSH secretion or modulate thyroid function (De Groef et al., 2006; Sower et al., 2009). This evolutionary divergence suggests that TRH may fulfill additional conserved functions outside the classical HPT axis, including roles in peripheral metabolic regulation.

Zebrafish (*Danio rerio*) represent a widely used vertebrate model for metabolic research due to their genetic accessibility, conserved endocrine systems, and physiological relevance to human metabolism (Espino-Saldaña et al., 2020; Zang et al., 2018). In this species, insulin signaling pathways are highly conserved relative to mammals (Emfinger et al., 2017, 2019); however, teleost-specific genome duplication has generated functionally specialized paralogs of key metabolic genes. Two insulin genes, *ins-a* and *ins-b*, are present, with *ins-a* representing the predominant and functionally relevant isoform during adult stages (Mullapudi et al., 2018). Likewise, two insulin receptor paralogs, *insr-a* and *insr-b*, exhibit distinct metabolic roles, with *insr-a* primarily associated with glucose utilization and lipogenic pathways, whereas *insr-b* contributes preferentially to lipid metabolism and protein synthesis (Gong et al., 2018; Yang et al., 2018). Glucose transport in zebrafish involves the transporters *slc2a2* (GLUT2) and *slc2a12* (GLUT12) (Marín-Juez et al., 2015; Houbrechts et al., 2019), which display tissue-specific expression patterns and support glucose sensing in liver and pancreas; notably, *slc2a12* has been proposed to mediate insulin-dependent glucose uptake in peripheral tissues (Castillo et al., 2009; Jiménez-Amilburu et al., 2015; Tseng et al., 2009).

Importantly, glucose homeostasis depends on the coordinated contribution of multiple organs, including the pancreas, liver, muscle, and brain, which integrate neuroendocrine signals with local metabolic responses (Schlegel and Gut, 2015; Zang et al., 2018). Growing evidence indicates that these regulatory mechanisms exhibit pronounced sexual dimorphism, with males and females displaying distinct transcriptional and physiological responses in metabolically active tissues (Battiprolu et al., 2007; Robison et al., 2008; Schuppe et al., 2017; Zhai et al., 2022). In models of metabolic stress, both sexes develop glycemic alterations, yet the magnitude and temporal dynamics of these responses differ, in association with sex-dependent regulation of insulin signaling pathways (Silva et al., 2024; Benchoula et al., 2024). Notably, sex-dependent metabolic effects of TRH have also been reported in mammalian systems (Ao et al., 2005; Bacová et al., 2005).

Within this context, identifying neuroendocrine systems capable of integrating tissue-specific and sex-dependent metabolic regulation is of particular relevance. Although TRH does not appear to act as a primary regulator of the HPT axis in zebrafish, other functions, such as the regulation of glucose homeostasis, may be conserved through its actions in peripheral tissues. While previous studies have largely focused on pancreatic TRH signaling, the liver represents a central metabolic hub for glucose sensing, storage, and insulin responsiveness (Tokarz et al., 2018), suggesting that TRH may modulate transcriptional components of insulin signaling in both pancreatic and hepatic tissues. Although TRH expression and function have been examined in other teleost species (Abbott and Volkoff, 2011; Bu et al., 2025; Feng et al., 2022), to date, the developmental expression, tissue-specific distribution, and sex-dependent metabolic roles of the TRHergic system in zebrafish remain unexplored. This knowledge gap enables an integrated investigation of the TRHergic system, combining bioinformatic analyses of conserved structural features with developmental and adult tissue-specific expression profiling, to assess whether TRH contributes to glucose homeostasis in a tissue- and sex-dependent manner in zebrafish.

## RESULTS

### Structural features of pre proTrh, Trh-rs, and Trh-de in zebrafish

**Fig. 1.**
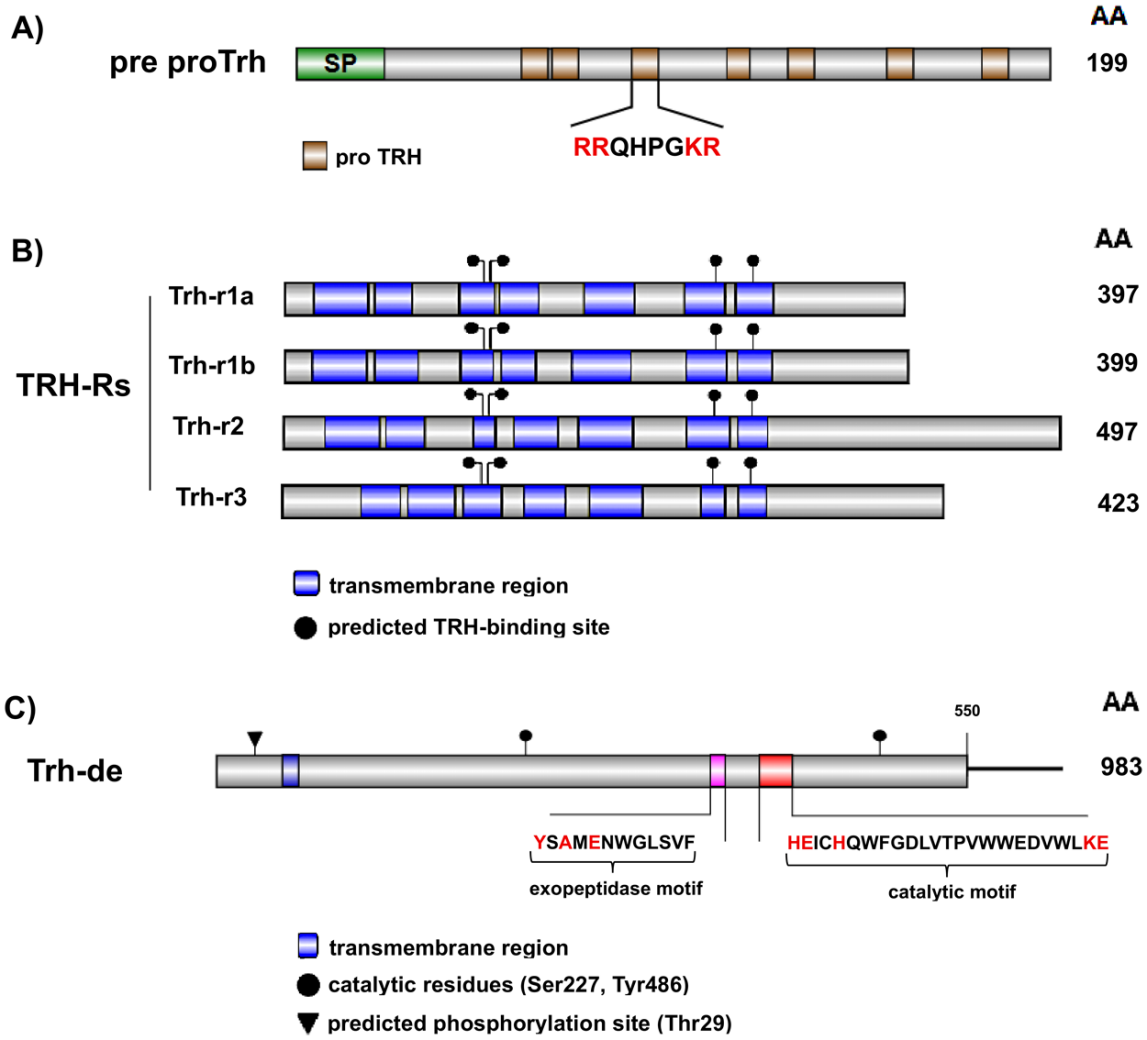
**Schematic representation of the molecular organization of the TRHergic system in zebrafish.** (A) pre proTrh schematic showing the signal peptide (SP, green) and multiple copies of the TRH progenitor sequence (proTRH, brown), each containing the conserved QHPG motif flanked by basic cleavage residues (RR and KR, red). (B) TRH-Rs, including Trh-r1a, Trh-r1b, Trh-r2, and Trh-r3, showing the predicted seven-transmembrane topology (blue) and the position of putative TRH-binding sites (black circles) inferred based on conserved residues previously reported (Laakkonen et al., 1996; Perlman et al., 1996). (C) Trh-de schematic illustrating the transmembrane domain (blue), the exopeptidase motif (pink), and the HEXXHX_18_E catalytic motif (red). Amino acids shown as black circles (Ser227 and Tyr486) and residues highlighted in red in the displayed sequences indicate key amino acid residues involved in TRH binding and catalysis. A predicted phosphorylation site (Thr29), potentially regulated by protein kinase C, is marked with a black arrowhead. For all proteins, the total amino acid length (AA) is indicated on the right.

Previous studies have conducted comprehensive phylogenetic analyses of pre proTRH, TRH-Rs, and TRH-DE across vertebrates, including zebrafish (Bu et al., 2025; Lazcano et al., 2021; Mekuchi et al., 2011; Saito et al., 2011). Accordingly, here we summarize the structural organization of these proteins in zebrafish, highlighting their conserved functional regions and putative amino acid residues inferred from sequence alignments (see Figs S1–S4) (Fig. 1). The zebrafish pre proTrh precursor consists of 199 amino acids and contains seven copies of the conserved Trh progenitor sequence (proTrh; QHPG). Each of these sequences is flanked by basic amino acid residues, lysine (K), and arginine (R), which serve as canonical cleavage sites essential for the release of mature TRH (Fig. 1A) (Joseph-Bravo et al., 2016; Wallis, 2010). Moreover, the predicted Trh-rs comprise four isoforms (Trh-r1a, Trh-r1b, Trh-r2, and Trh-r3), each retaining the characteristic seven transmembrane domains of GPCRs and conserved residues involved in TRH binding. The inferred ligand-interacting residues include Tyr105 and Asn109 in TM3, Tyr276 in TM6, and Arg300 in TM7 for Trh-r1a; Tyr103, Asn107, Tyr277, and Arg301 for Trh-r1b; Tyr112, Asn116, Tyr287, and Arg311 for Trh-r2; and Tyr153, Asn157, Tyr324, and Arg348 for Trh-r3 (positions deduced by homology with murine TRH-Rs; Laakkonen et al., 1996; Perlman et al., 1996) (Fig. 1B). Lastly, the zebrafish Trh-de exhibits the canonical structural motifs of M1 zinc-dependent metallopeptidases, including a predicted transmembrane region, an exopeptidase motif (AMENWGLSVF), and the catalytic HEXXHX₁₈E signature (Fig. 1C) (Bauer, 1994; Charli et al., 1998). Functionally relevant residues predicted to participate in TRH substrate recognition include Ser227, Tyr361, Ala363, Glu365, and Lys421, while essential catalytic residues are His399, Glu400, His403, Glu422, and Tyr486. Additionally, a potential intracellular phosphorylation site (Thr29) was identified, which has been proposed to be regulated by protein kinase C. Collectively, these structural features confirm the conserved organization and functional domains of the zebrafish TRHergic components, consistent with their vertebrate counterparts.

### Ontogeny of *trh*, *trh-rs*, and *trh-de* expression in zebrafish

**Fig. 2.**
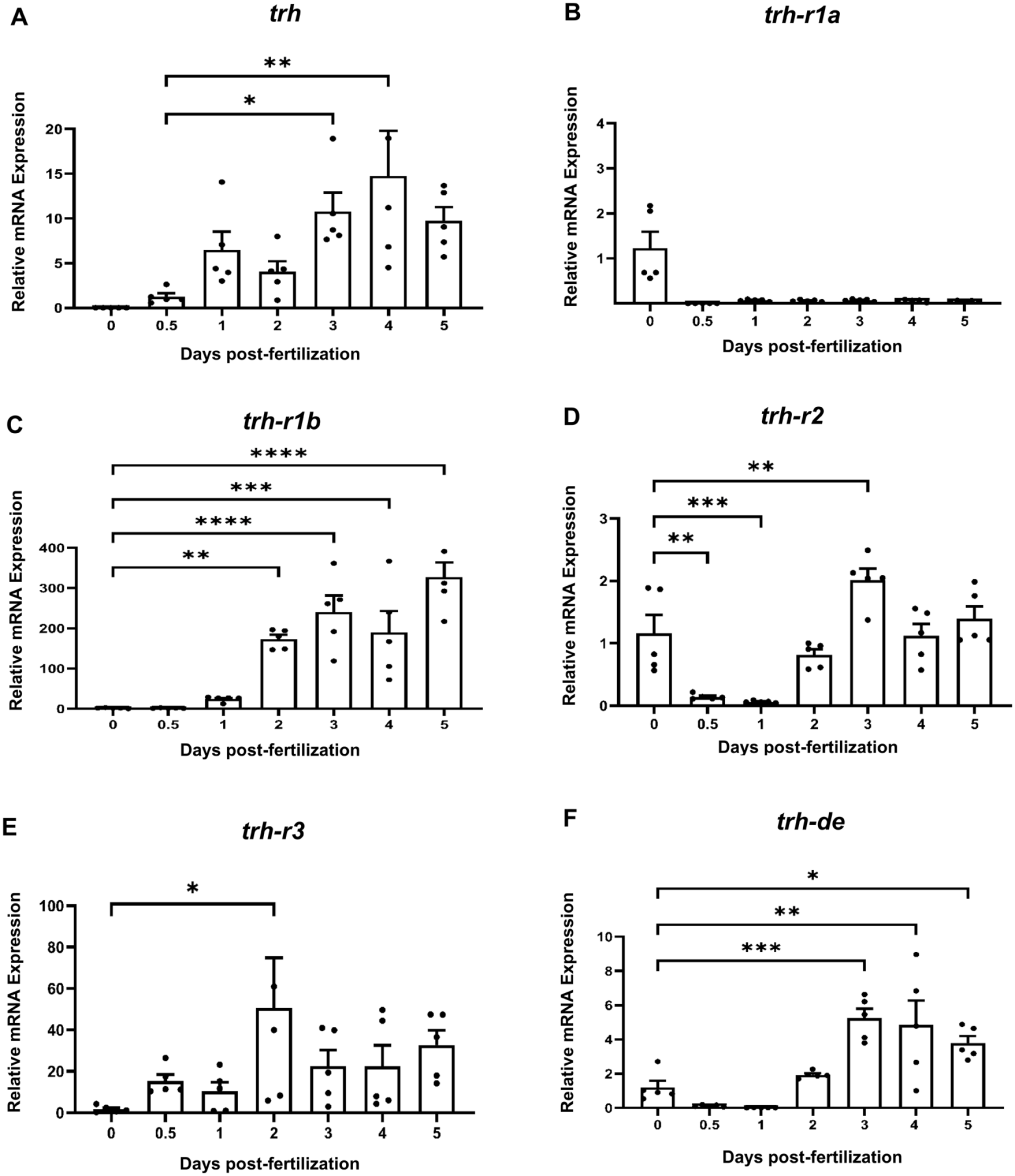
**Relative expression of *trh*, *trh-r* isoforms, and *trh-de* during early zebrafish development.** (A–F) Relative mRNA expression of *trh* (A), *trh-r1a* (B), *trh-r1b* (C), *trh-r2* (D), *trh-r3* (E), and *trh-de* (F) was quantified from 0 to 5 dpf. Expression values were normalized to two reference genes (*18S* rRNA and *lsm12b*) and are presented as mean±s.e.m. Expression at each developmental stage was compared with 0 dpf, except for *trh*, which was normalized to 0.5 dpf (12 hpf) due to undetectable transcript levels at fertilization. For each time point, *n*=5 biological replicates were analyzed, with each sample measured in technical duplicate. Statistical analysis was performed using one-way ANOVA followed by Tukey's multiple comparisons test (two-tailed). Statistical significance indicates differences relative to the corresponding reference stage: *P<0.05, **P<0.01, ***P<0.001, ****P<0.0001. Data are representative of one independent experiment using biological replicates.

Expression analysis of the key elements involved in TRH signaling was conducted to correlate their expression during early zebrafish development. The expression of *trh* was undetectable at fertilization [0 days post fertilization (dpf)] but became detectable at 0.5 dpf [12 h post fertilization (hpf)], followed by a gradual and significant increase at 3 dpf that remained elevated in the following days (*P*<0.05) (Fig. 2A). Among the receptor isoforms, *trh-r1a* remained practically absent throughout the analyzed period (Fig. 2B), while *trh-r1b* showed the most remarkable and sustained upregulation, with a pronounced increase from 2 dpf onward, reaching an expression level approximately 300-fold higher at 5 dpf relative to its expression at 0 dpf (*P*<0.0001) (Fig. 2C). *trh-r2* maintained relatively low expression but displayed a significant increase at 3 dpf (*P*<0.01) (Fig. 2D). The *trh-r3* isoform exhibited a transient induction, with a significant increase at 2 dpf (*P*<0.05), followed by a return to lower and relatively stable expression levels at later stages (3–5 dpf), without further statistically significant changes (Fig. 2E). Finally, *trh-de* expression was minimal during early developmental stages but increased significantly from 3 dpf onward (*P*<0.001), remaining elevated at later stages (Fig. 2F).

### Tissue- and sex-specific expression of *trh*, *trh-r*, and *trh-de* in zebrafish

**Fig. 3.**
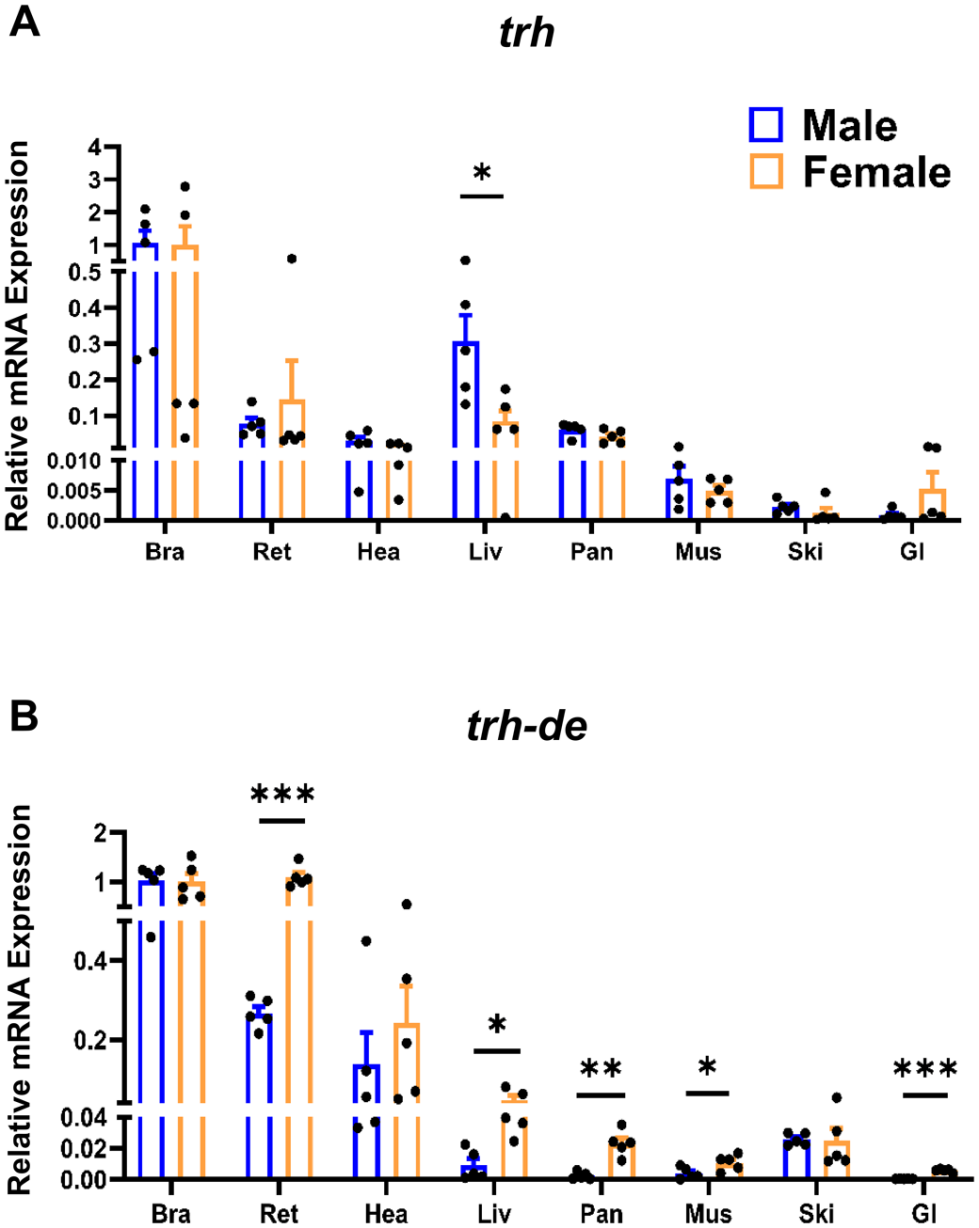
**Relative expression of *trh* and *trh-de* in adult zebrafish tissues.** (A,B) Relative mRNA expression of *trh* (A) and *trh-de* (B) in central and peripheral tissues from adult male and female zebrafish (Bra, brain; Ret, retina; Hea, heart; Liv, liver; Pan, pancreas; Mus, muscle; Ski, skin; GI, gastrointestinal tract). Gene expression was normalized to the brain as the reference tissue using *β-actin* and *18S* rRNA as housekeeping genes. Bars represent mean±s.e.m. Blue and orange bars correspond to males and females, respectively. For each tissue, *n*=5 biological replicates were analyzed; individual tissues were processed separately, except for pancreas and heart, which were analyzed as pools of three tissues per replicate. Sex differences within each tissue were analyzed using unpaired two-tailed Student's *t*-test. Statistical significance is indicated as follows: *P<0.05, **P<0.01, ***P<0.001. Data are representative of one independent experiment using biological replicates.

The analysis of *trh*, *trh-rs*, and *trh-de* expression across multiple zebrafish tissues revealed marked tissue-specific and sex-dependent patterns. In general, *trh* expression in the brain was more than ten times greater than that observed in the retina, heart, liver, and pancreas (Fig. 3A), with the latter two being key organs involved in glucose homeostasis. Also, lower *trh* transcript levels were detected in the skin and gastrointestinal (GI) tract. With respect to sex differences, a significant dimorphism was observed only in the liver, where *trh* expression was higher in males (mean ∼0.31) compared with females (∼0.08). Mean values are provided to aid visualization of differences that may be less apparent in the graphical representation.

**Fig. 4.**
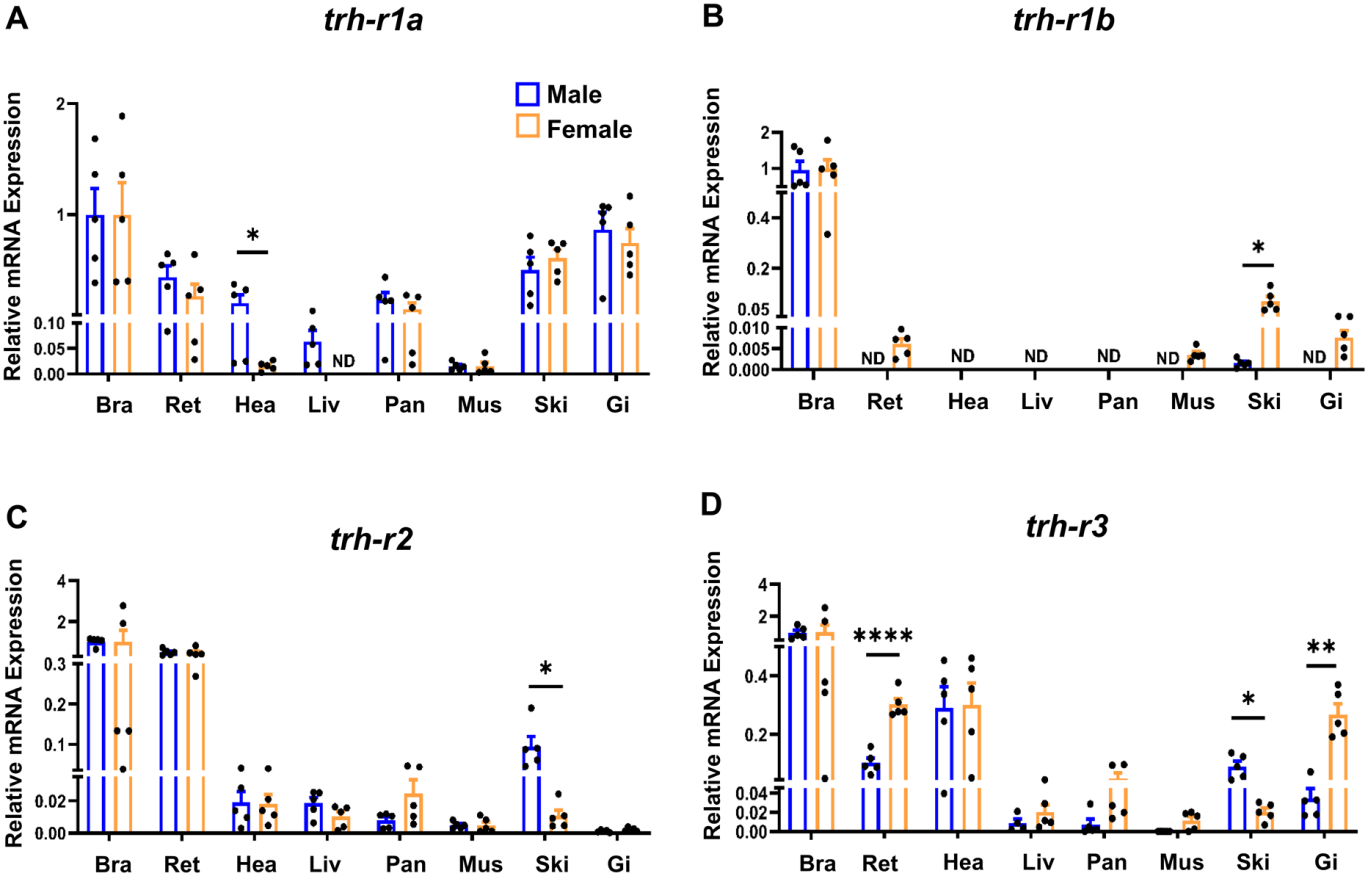
**Relative expression of *trh-r* isoforms in adult zebrafish tissues.** (A–D) Relative mRNA expression levels of *trh-r1a* (A), *trh-r1b* (B), *trh-r2* (C), and *trh-r3* (D) were quantified in central and peripheral tissues from adult male and female zebrafish (Bra, brain; Ret, retina; Hea, heart; Liv, liver; Pan, pancreas; Mus, muscle; Ski, skin; GI, gastrointestinal tract). Gene expression was normalized to the brain as the reference tissue, using *β-actin* and *18S* rRNA as housekeeping genes. Bars represent mean±s.e.m. Blue and orange bars correspond to males and females, respectively. For each tissue, *n*=5 biological replicates were analyzed; individual tissues were used except for pancreas and heart, which were processed as pools of three tissues per replicate. Sex differences within each tissue were analyzed using unpaired two-tailed Student's *t*-test. Statistical significance is indicated as follows: *P<0.05, **P<0.01, ****P<0.0001. ND indicates transcripts not detected. Data are representative of one independent experiment using biological replicates.

As observed for *trh*, expression of the four genes encoding *trh-rs* reached their highest transcript abundance in the brain and retina (Fig. 4A–D), where all receptor genes displayed comparable expression levels, with detectable expression also observed in several peripheral organs. Among the receptors, *trh-r1a* was more highly expressed in non-brain tissues than the other *trh-r* genes studied and was detected in all peripheral tissues examined, with notable levels in the heart, liver, pancreas, skin, and GI tract (Fig. 4A). A marked sex-dependent difference was observed in the heart, where *trh-r1a* expression was substantially higher in males (∼0.20) than in females (∼0.015). In the liver, *trh-r1a* transcripts were detected exclusively in males. In contrast, *trh-r1b* exhibited low expression across the tissues analyzed and was not detectable in the heart, liver, and pancreas (Fig. 4B). Female skin, however, showed significantly higher *trh-r1b* expression, indicating a possible sex-specific function. The *trh-r2* transcript displayed a distribution pattern comparable to that of *trh-r1a* in the brain and retina but showed lower expression levels in peripheral tissues (Fig. 4C). The expression of this receptor was higher in male skin (∼0.095 versus ∼0.011 in females). *trh-r3* was expressed in the brain and retina of both sexes, with relatively higher expression levels in the heart and lower transcript abundance across the remaining peripheral tissues (Fig. 4D). Sex-related differences included higher expression in the retina and GI tract of females (∼0.27 vs ∼0.035 in males), as well as increased expression in the skin of males (∼0.09 versus ∼0.02 in females).

Consistent with the distribution of other TRH pathway components, *trh-de* expression was more abundant in the brain and retina (Fig. 3B) and was detectable in all peripheral tissues. Significant sex-dependent differences were detected in the retina and liver, as well as in peripheral tissues where expression was less readily appreciable in the graphs, including the pancreas (∼0.023 versus ∼0.002), muscle (∼0.011 versus ∼0.004), and the GI tract (∼0.0056 versus ∼0.0004), with females exhibiting higher expression levels in all cases.

### Exogenous TRH lowers glucose levels in adult male zebrafish

After assessing the expression patterns of TRH signaling components in central and peripheral tissues, the presence of these elements in metabolically active organs prompted the evaluation of TRH's potential role in glycemic regulation. To this end, adult male and female zebrafish were subjected to a hyperglycemic model and received daily intraperitoneal (ip) TRH injections for three consecutive days (Fig. S9).

**Fig. 5.**
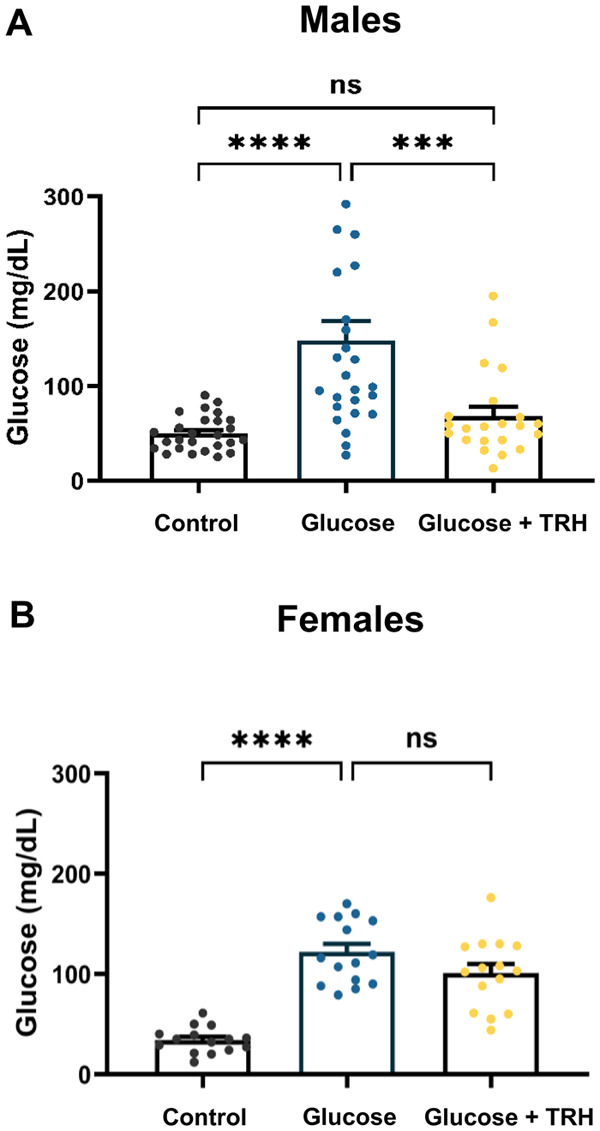
**Effect of TRH on blood glucose levels in hyperglycemic adult zebrafish.** (A,B) Male (A) and female (B) zebrafish were subjected to an experimental model of induced hyperglycemia and treated with daily ip injections of 1 µg of TRH for three consecutive days. Blood glucose levels were assessed in three groups: control+saline (sham), glucose+saline (hyperglycemic), and glucose+TRH (hyperglycemic, TRH-treated). Data are presented as mean±s.e.m. For each experimental group, *n*=15 adult specimens were analyzed. Statistical analysis was performed using one-way ANOVA followed by Tukey's multiple comparisons test (two-tailed). Statistical significance is indicated as follows: ***P<0.001, ****P<0.0001; ns, not significant. Data are representative of one independent experiment.

In male zebrafish (Fig. 5A), induction of hyperglycemia resulted in a significant elevation of blood glucose levels (161.7±10.2 mg/dl) compared to the control group (52.8±3.4 mg/dl). Notably, TRH administration induced a substantial reduction in glucose levels (76.9±5.8 mg/dl), representing an approximate 53% decrease relative to the hyperglycemic group. Importantly, glucose levels in the TRH-treated group did not differ significantly from those of the control group, indicating a recovery toward baseline glycemia. These values are consistent with our observations of non-fasted glucose concentrations in adult zebrafish (60–80 mg/dl; Fig. S8).

In female zebrafish (Fig. 5B), the hyperglycemic protocol similarly led to a significant elevation in blood glucose (111.3±5.6 mg/dl) relative to controls (49.2±2.9 mg/dl). However, TRH administration did not elicit a statistically significant reduction in glucose levels (106.7±6.1 mg/dl), suggesting a sex-dependent difference in the glycemic response to TRH under the same experimental conditions.

Additionally, in an independent experiment using the hyperglycemia model in a small cohort of male zebrafish (*n*=6), administration of human insulin resulted in a significant reduction in blood glucose levels, decreasing from 79.2±20.2 mg/dl in glucose-exposed fish to 48.3±7.9 mg/dl following insulin treatment. This result confirms the responsiveness of the model to a canonical insulin-mediated stimulus (Fig. S10).

### Differential transcriptional responses to TRH and hyperglycemia reveal sex-dependent metabolic regulation in adult zebrafish

To further explore the molecular mechanisms underlying the glycemic responses observed after TRH administration in the hyperglycemia model, we quantified the expression of glucose metabolism-related genes and TRHergic genes in the pancreas and liver of adult male and female zebrafish.

**Fig. 6.**
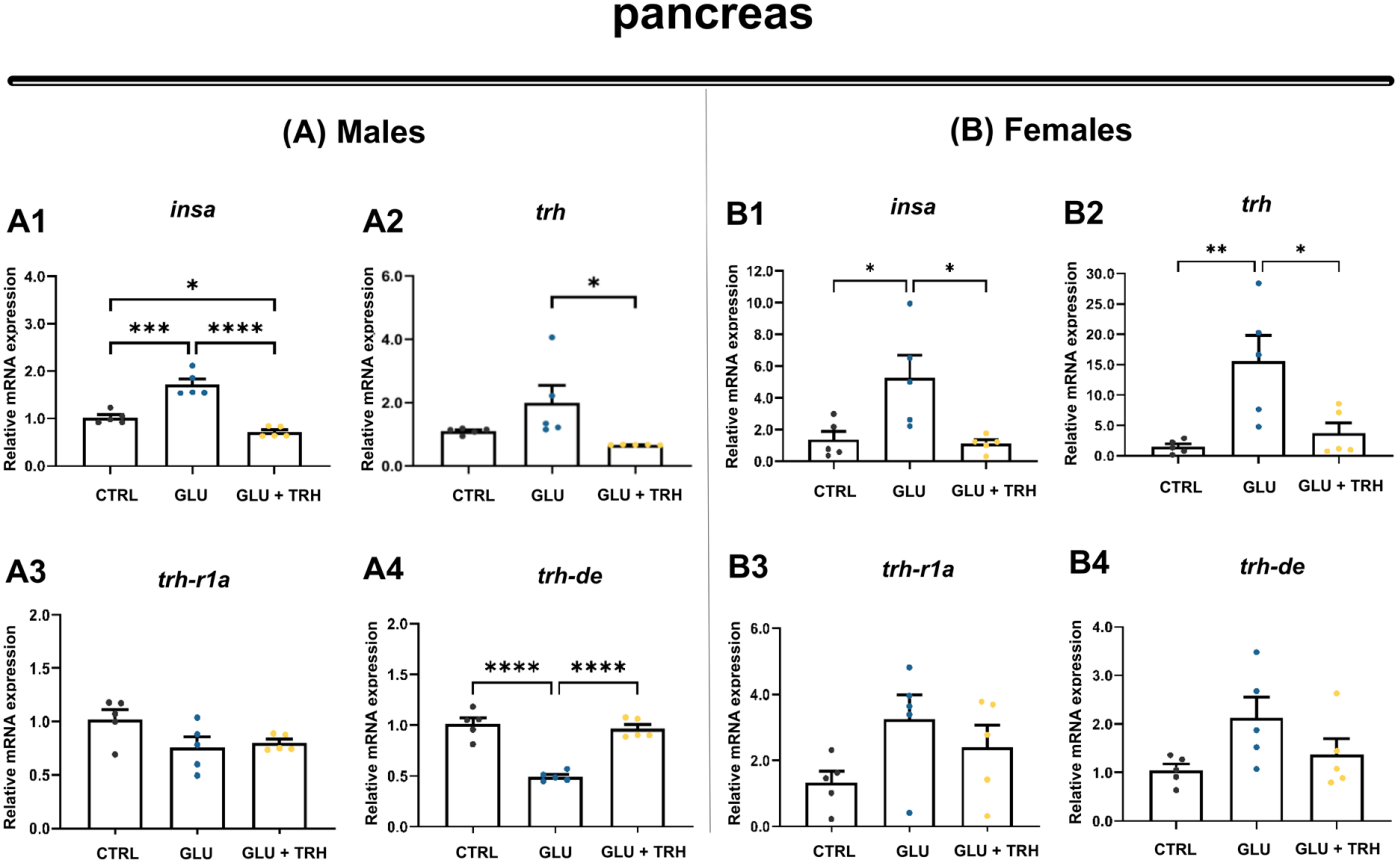
**Relative mRNA expression of insulin and TRHergic components in the pancreas of adult male and female zebrafish.** (A1–B4) Relative expression of *insa* and TRHergic system components (*trh*, *trh-r1a*, and *trh-de*) was quantified in pancreatic tissue from adult zebrafish under three experimental conditions: control (vehicle-injected; CTRL), glucose (hyperglycemic, vehicle-injected; GLU), and glucose+TRH (hyperglycemic, treated with TRH at 1 µg/g BW; GLU+TRH). Panels A1–A4 correspond to males and B1–B4 correspond to females, illustrating sex-dependent transcriptional responses for each gene: *insa* (A1,B1), *trh* (A2,B2), *trh-r1a* (A3,B3), and *trh-de* (A4,B4). Each biological replicate consisted of a pool of three pancreas per sample. Gene expression levels were normalized to *β-actin* and *18S* rRNA and are presented as mean±s.e.m. (*n*=5 per group). Statistical comparisons across treatments were performed within each sex using one-way ANOVA followed by Tukey's multiple comparisons test (two-tailed). Significant differences are indicated as follows: *P<0.05, **P<0.01, ***P<0.001, ****P<0.0001. Data are representative of one independent experiment using biological replicates.

As expected for a compensatory response to hyperglycemia, *insa* was strongly upregulated relative to controls (*P*<0.001) (Fig. 6A1). TRH treatment significantly reduced *insa* expression, reaching levels even lower than those observed under control conditions (*P*<0.05). Pancreatic *trh* expression remained unchanged during hyperglycemia but decreased significantly following TRH exposure (*P*<0.05) (Fig. 6A2). Expression of *trh-r1a* was not significantly affected by either sustained glucose exposure or TRH treatment (Fig. 6A3). In contrast, *trh-de* expression was markedly significantly downregulated under glucose treatment (*P*<0.0001) and returned to near-control levels following TRH administration (Fig. 6A4).

In females, hyperglycemia similarly induced an upregulation of pancreatic *insa* expression (*P*<0.05) (Fig. 6B1), while TRH treatment restored *insa* levels to those observed in controls. Among TRHergic components, hyperglycemia significantly increased pancreatic *trh* expression, an effect that was reversed by TRH treatment, restoring *trh* levels comparable to controls (Fig. 6B2). In contrast, neither *trh-r1a* nor *trh-de* expression was significantly affected by glucose exposure or TRH treatment (Fig. 6B3,B4).

**Fig. 7.**
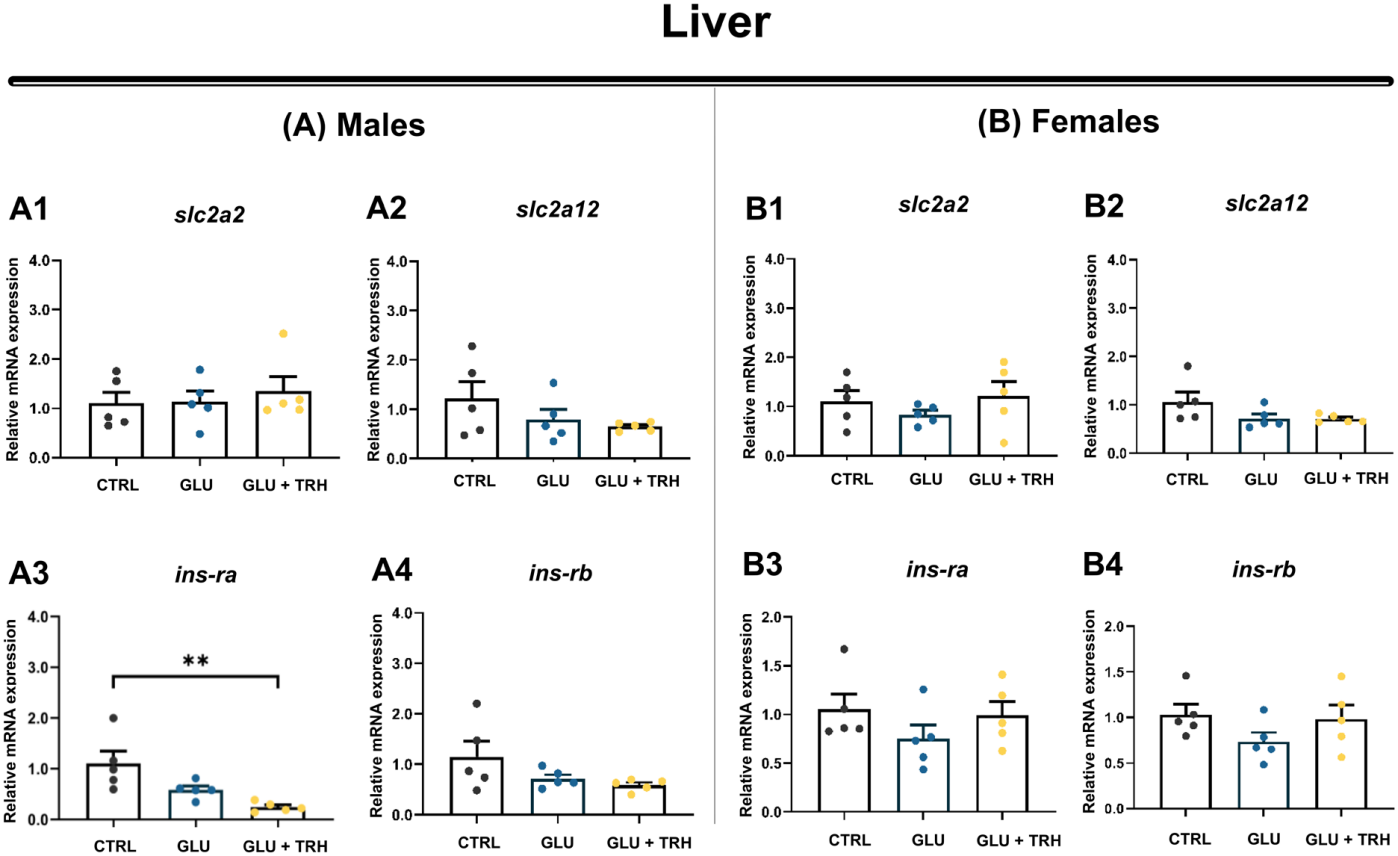
**Relative mRNA expression of glucose transporters and insulin receptor isoforms in the liver of adult male and female zebrafish.** (A1–B4) Relative expression of the glucose transporters *slc2a2* and *slc2a12*, together with the insulin receptor isoforms *ins-ra* and *ins-rb*, was quantified in liver tissue from adult zebrafish exposed to three experimental conditions: control (vehicle-injected; CTRL), glucose (hyperglycemic, vehicle-injected; GLU), and TRH (hyperglycemic, treated with TRH at 1 µg/g BW; GLU+TRH). Panels A1–A4 show expression patterns for males, and B1–B4 for females, corresponding to *slc2a2* (A1,B1), *slc2a12* (A2,B2), *ins-ra* (A3,B3), and *ins-rb* (A4,B4). Gene expression levels were normalized to *β-actin* and *18S* rRNA and are presented as mean±s.e.m. (*n*=5 per group). Statistical comparisons across treatments were performed within each sex using one-way ANOVA followed by Tukey's multiple comparisons test (two-tailed). Significant differences are indicated as follows: **P<0.01. Data are representative of one independent experiment using biological replicates.

In the male liver, sustained hyperglycemia did not significantly alter the expression of glucose metabolism-related genes (Fig. 7A1–A4). However, TRH treatment selectively reduced *ins-ra* expression (*P*<0.01), while the expression of glucose transporter isoforms and *ins-rb* remained unchanged (Fig. 7A1–A4). In females, hepatic expression of glucose metabolism–related genes was not significantly affected by either hyperglycemia or TRH treatment (Fig. 7B1–B4).

**Fig. 8.**
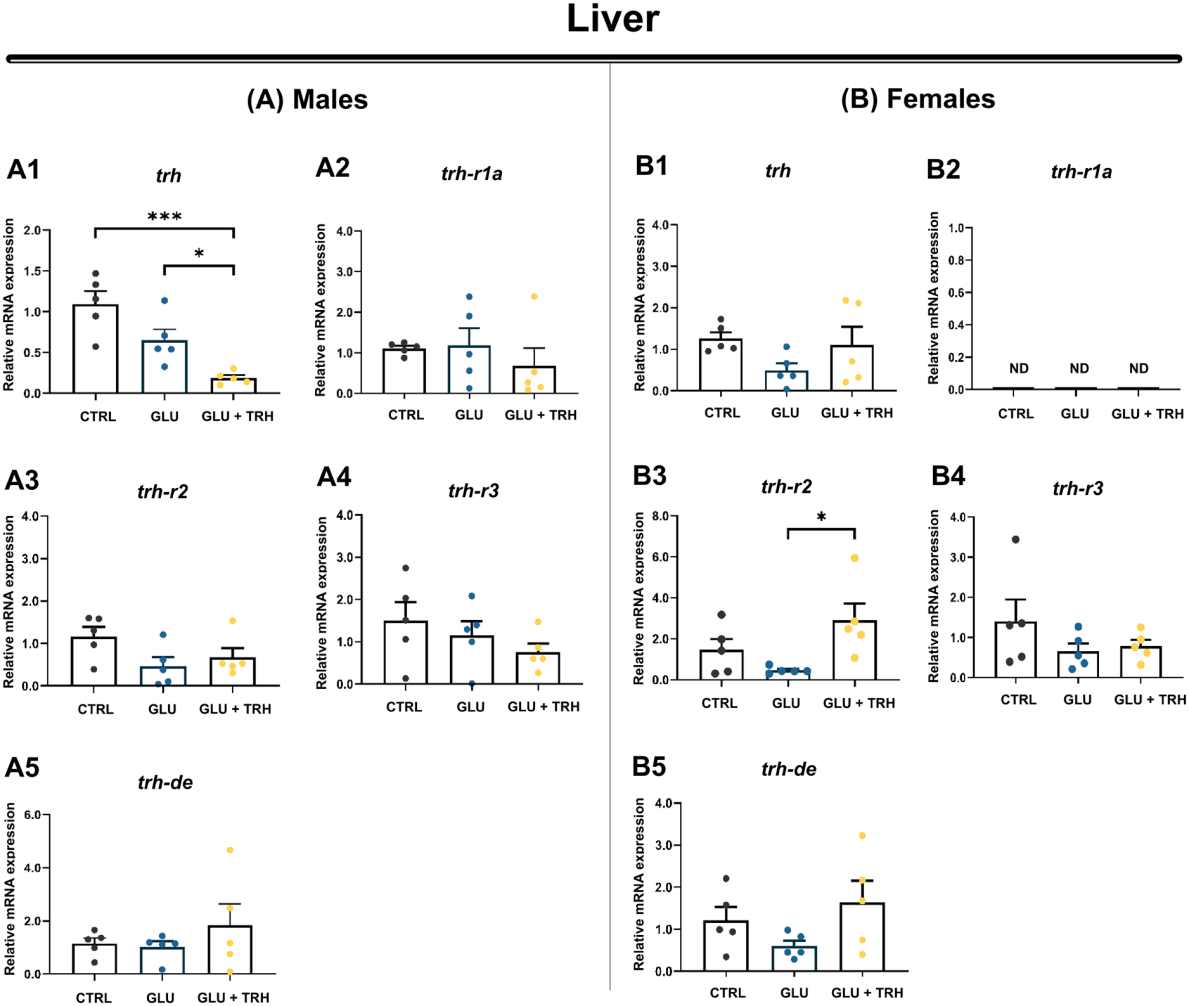
**Relative mRNA expression of TRHergic system components in the liver of adult male and female zebrafish.** (A1–B5) Hepatic expression of *trh*, *trh-r1a*, *trh-r2*, *trh-r3*, and *trh-de* was quantified in adult zebrafish subjected to three experimental conditions: control (vehicle-injected; CTRL), glucose (hyperglycemic, vehicle-injected; GLU), and TRH (hyperglycemic, treated with TRH at 1 µg/g BW; GLU+TRH). Panels A1–A5 correspond to males and B1–B5 to females, illustrating sex-dependent hepatic expression patterns for *trh* (A1,B1), *trh-r1a* (A2,B2), *trh-r2* (A3,B3), *trh-r3* (A4,B4), and *trh-de* (A5,B5). In females, *trh-r1a* transcripts were not detected across experimental groups (ND). Each biological replicate consisted of an individual liver sample (*n*=5 per group). Gene expression levels were normalized to *β-actin* and *18S* rRNA and are presented as mean±s.e.m. Statistical comparisons across treatments were performed within each sex using one-way ANOVA followed by Tukey's multiple comparisons test (two-tailed). Significance is indicated as follows: *P<0.05, ***P<0.001. Data are representative of one independent experiment using biological replicates.

Analysis of TRHergic components in the male liver revealed a significant reduction in hepatic *trh* expression under both hyperglycemic conditions and following TRH treatment compared with controls (*P*<0.05 and *P*<0.001, respectively) (Fig. 8A1). In contrast, the expression of the remaining TRHergic components remained unchanged across treatments (Fig. 8A2–A5).

In females (Fig. 8B1–B5), hepatic *trh* expression was not significantly affected by glucose exposure or TRH treatment (Fig. 8B1). The receptor *trh-r1a* was evaluated; however, its expression remained undetectable in the female liver across all experimental groups (Fig. 8B2), consistent with our adult tissue characterization, in which females likewise showed no detectable levels of this receptor. Overall, most TRHergic components did not exhibit significant transcriptional changes under hyperglycemic conditions or following TRH administration. Notably, TRH treatment induced a significant upregulation of *trh-r2* expression (*P*<0.05), suggesting a selective, sex-specific modulation of this receptor in the female liver (Fig. 8B3).

## DISCUSSION

### Evolutionary conservation and structural integrity of the zebrafish TRHergic system

TRH is a highly conserved neuropeptide that regulates endocrine, metabolic, and behavior in vertebrates (Alvarez-Salas et al., 2022; Joseph-Bravo et al., 2015; Lechan and Fekete, 2006; Vargas et al., 2024). The core components of TRH signaling, including the TRH peptide, its receptors (TRH-Rs), and the TRH-degrading enzyme (TRH-DE), retain key structural domains essential for their biological activity across vertebrate evolution (Galas et al., 2009; Lazcano et al., 2021, 2025). Consistent with previous comparative studies (Bu et al., 2025; Lazcano et al., 2021; Mekuchi et al., 2011; Saito et al., 2011), our structural analyses confirm that zebrafish TRHergic components preserve the canonical molecular organization described in other vertebrates, including conserved QHPG motifs in pre proTRH, GPCR features in TRH receptor isoforms implicated in ligand recognition, and catalytic signatures characteristic of M1 zinc-dependent metallopeptidases in TRH-DE. Together, these findings establish a conserved molecular framework that suggests a conserved physiological role. Accordingly, we integrate ontogenetic, tissue-specific, and sex-dependent expression patterns with functional analyses under metabolic challenge to further characterize the zebrafish TRHergic system.

### Ontogenetic activation of TRHergic signaling during early development

The developmental dynamics of the TRHergic system provide insight into how this signaling pathway becomes functionally engaged during early life stages in this model. Notably, no evidence of maternal transcript contribution was detected for TRHergic signaling genes; however, most components exhibited a progressive increase in expression from 1 to 5 dpf, suggesting that TRH signaling could be activated zygotically and could participate in early developmental processes.

Consistent with this pattern, *trh* expression gradually increased from 0.5 to 4 dpf, coinciding with CNS development and the maturation of neuroendocrine axes (Lazcano et al., 2023). Although TRH does not regulate the activation of the HPT axis in fish, it has been shown to induce the release of growth hormone (GH) and α-melanocyte stimulating hormone (α-MSH), suggesting that it may be involved in the activation of other neuroendocrine axes (Galas et al., 2009; Lazcano et al., 2025).

Among the receptors, *trh-r1b* exhibited a pronounced expression peak during this period, suggesting a prominent role in early TRH signaling. This finding is consistent with previous reports in zebrafish and *Pimephales promelas*, which also reveal early expression of this receptor (Vergauwen et al., 2018). In contrast, *trh-r3* showed a transient rise at 2 dpf, whereas *trh-r1a* and *trh-r2* remained at low levels. Notably, *trh-r1a* has been shown to be specifically expressed in the hatching gland at 2 dpf in zebrafish, where TRH is required for its activation (Gajbhiye et al., 2024). In our study, *trh-r1a* presented very low expression levels, indicating that the critical tissue-specific roles for TRH-Rs may not be detectable when global expression profiles are analyzed in whole embryos. *Trh-de* expression was low and constant from 0 to 5 dpf, suggesting that this enzyme may be acting discreetly upon TRH degradation during early stages, favoring prolonged signaling during developmental processes.

### Central and peripheral distribution of TRHergic components in adult zebrafish

In adult zebrafish, the TRHergic signaling genes were detected in both the CNS and various peripheral tissues. As expected, the highest expression levels were observed in the brain, consistent with extensive TRH immunoreactivity reported across teleost species in regions such as the hypothalamus, preoptic area, telencephalon, midbrain, olfactory bulb, and brainstem (Batten et al., 1990; Buckley et al., 2010; Díaz et al., 2002; Hamano et al., 1990). This distribution suggests a central neuromodulatory role in functions such as sensory integration, motor control, and the regulation of locomotor and feeding behaviors (Abbott and Volkoff, 2011; Rotllant et al., 2000; Singh et al., 2019; Vargas et al., 2024). As described in mammals, TRH signaling in the CNS is likely enriched in neuronal populations; accordingly, we do not exclude that zebrafish preserves a similar neuronal signaling network, which can be effectively investigated using this model (Joseph-Bravo et al., 2015; Müller-Fielitz et al., 2017; Sánchez et al., 2009).

Notably, after the brain, the retina exhibited one of the highest expression levels of TRHergic components in both sexes. This finding is consistent with previous reports demonstrating TRH, TRH receptors, and TRH-DE expression in the vertebrate retina. In teleosts and amphibians, TRH immunoreactivity has been primarily localized to amacrine cells (Anadón et al., 2002; Díaz et al., 2002), whereas in humans TRH and TRH-R are broadly distributed throughout the retina (Dubovy et al., 2017). Moreover, single-cell RNA sequencing studies in mammals have identified TRHergic signaling elements across multiple retinal cell types, including amacrine cells, photoreceptors, and astrocytes (Yan et al., 2020). Together, these observations suggest that the TRHergic system is present in the vertebrate retina and reinforce the utility of zebrafish as a model to study neuropeptidergic signaling in sensory tissues.

In addition, our analysis revealed relatively high *trh* expression in the liver, particularly in males. Although the liver is not considered a canonical site of *TRH* expression in mammals, where *TRH-Rs* and *TRH-DE* are more commonly detected (Charli et al., 2020; Mitsuma et al., 1995; Vargas et al., 2024), hepatic *TRH* expression has been reported in non-mammalian vertebrates, including teleosts (Feng et al., 2022; Ruiz-Jarabo et al., 2018) and reptiles (Ávila-Mendoza et al., 2018). In these models, hepatic *TRH* expression has been interpreted as supporting local autocrine or paracrine signaling rather than classical endocrine actions. The detection of both *trh-rs* and *trh-de* in the zebrafish liver suggests that this tissue may integrate TRH signaling in a physiological state-dependent manner, potentially contributing to local metabolic regulation.

Consistent with this peripheral distribution, TRHergic components were also detected in metabolically active organs such as the pancreas, GI tract, and heart, supporting a broader role for TRH signaling in energy homeostasis. In mammals, TRH has been implicated in the regulation of insulin secretion, GI motility, energy metabolism, and gastric secretory activity (Fekete and Lechan, 2014; Lechan and Fekete, 2006; Luo et al., 2014; Morley et al., 1977; Taché et al., 2006; Zhang et al., 2018b). Similarly, recent studies in teleosts have reported the expression of pre proTRH and its receptors in peripheral tissues, including muscle, skin, intestine, kidney, and liver (Blanco, 2020; Bu et al., 2025; Feng et al., 2022), reinforcing the concept of a conserved peripheral TRHergic system across vertebrates.

In particular, the presence of *trh* transcripts in pancreatic β cells has previously been reported in zebrafish through transcriptomic analyses (Tarifeño-Saldivia et al., 2017). In mammals, *TRH* expression and signaling have also been documented in pancreatic β cells (Basmaciogullari et al., 2000; Luo and Yano, 2004; Mulla et al., 2009), where TRH has been shown to influence hormonal regulation as well as the maturation and differentiation of pancreatic islets (Amir, 1988; Luo and Yano, 2004; Luo et al., 2008, 2014; Štrbák, 2018). Together, these observations highlight the possibility that locally produced TRH may influence β-cell physiology in zebrafish.

Skin was also included as a tissue of interest given its recognized role as a specialized neuroendocrine organ in vertebrates (Slominski and Wortsman, 2000). In amphibians such as *Rana pipiens*, TRH is highly abundant in the skin and has been proposed to function as a major peripheral reservoir, where it is localized to dermal granular glands and actively released in response to adrenergic stimulation, suggesting roles in osmoregulation and environmental adaptation (Bennett et al., 1981; Jackson and Reichlin, 1977). In humans, TRH and its receptor have been detected in hair follicles, where TRH exerts direct functional effects by stimulating hair growth, prolonging the anagen phase, and reducing apoptosis of hair matrix cells (Gáspár et al., 2010). Although the presence of TRH in teleost skin has been previously reported (Ruiz-Jarabo et al., 2018), functional evidence remains limited; nevertheless, the detection of TRHergic components in this tissue suggests potential peripheral neuroendocrine roles that warrant further investigation.

In parallel, the widespread expression of *trh-de* in both central and peripheral tissues suggests that local TRH availability may be tightly regulated through enzymatic degradation. In mammals, a hepatic isoform of TRH-DE has been described that participates in the inactivation of circulating TRH (Schmitmeier et al., 2002; Charli et al., 2020; Fröhlich and Wahl, 2019). In the present study, the enzymatic activity of trh-de on circulating TRH could not be evaluated due to limitations in the availability of zebrafish serum samples.

### Sexual dimorphism in the TRHergic system

Sexual dimorphism represents a major source of physiological variability in fish and can significantly influence endocrine and metabolic regulation (Campbell et al., 2021; Li et al., 2024; Zhou et al., 2021). In zebrafish, distinct sex-specific patterns have been described, indicating that males and females adopt differential physiological and metabolic strategies. For example, transcriptomic analyses of metabolically active tissues such as the liver and heart have consistently reported higher expression of transcription factors linked to energy metabolism in males, supporting a metabolic profile indicative of increased energetic activity (Abu Nahia et al., 2024; Robison et al., 2008; Zhang et al., 2018a; Zheng et al., 2013). Concordantly, males exhibit a more robust activation of the stress axis and enhanced cortisol-dependent energy mobilization, which translates into faster metabolic responses to environmental challenges (Goikoetxea et al., 2017). At the physiological level, males show a higher basal metabolic rate during juvenile stages (∼3 months), which later declines and becomes lower than that of females at older ages (≥1 year) (Konadu et al., 2023). In contrast, females exhibit a more active lipid metabolism and a greater tendency toward energy accumulation and storage, together with increased protein synthesis and cellular growth (Robison et al., 2008), accompanied by increased sensitivity to sensory stimuli and more efficient neuroendocrine integration (Genario et al., 2020; Gorissen and Flik, 2016).

Our findings suggest a sex-dependent regulation of the TRHergic system. In males, the elevated hepatic expression of *trh* and the higher expression of *trh-r1a* in the heart partially align with transcriptomic evidence indicating increased activity of metabolic- and energy-related gene programs in these tissues. Together, these observations point to tissue-specific regulatory mechanisms, with *trh* enrichment in the liver and elevated *trh-r1a* expression in the heart indicating distinct potential sites of TRH action. Through receptor-mediated signaling, TRH may therefore modulate metabolic and functional processes in a tissue-dependent manner, potentially exerting more pronounced effects in males. Notably, functional evidence supports a role for TRH in the heart in mammalian models (Peres Díaz et al., 2018). In addition to this sexual dimorphism, the observed patterns may be further influenced by the higher metabolic rate reported in males during juvenile stages, which partially overlaps with the age range of the organisms analyzed in this study (3–12 months).

Conversely, females exhibited selective sex-dependent features within the TRHergic system, characterized by higher expression of *trh-r3* in the GI tract and consistently elevated *trh-de* expression across multiple tissues. The increased expression of *trh-de* suggests a tighter modulation of TRH availability in females. Such regulatory features may be associated with processes related to energy handling, tissue maintenance, and reproductive physiology. Importantly, the presence of *TRH* and/or its receptors in the GI tract has been reported in several teleost species, including medaka (Mekuchi et al., 2011), tilapia (Bu et al., 2025), winter flounder (Buckley et al., 2010), and rice field eel (Feng et al., 2022), without explicit assessment of sex-specific differences or tissue-specific effects. In mammals, *TRH* expression in the GI tract has been proposed to function as a local regulatory hormone involved in the modulation of motility and hormonal secretion (Morley et al., 1977).

### Sex-dependent hypoglycemic effects of TRH under metabolic challenge

In a hyperglycemic model, we found that TRH administration significantly reduced circulating glucose levels exclusively in adult males, supporting the notion that TRH is bioactive under metabolic challenge and may contribute to glucose regulation in a sex-dependent manner. Sex-specific actions of TRH on glucose homeostasis have also been described in mammalian models. Ao et al. (2005) reported that central administration of a low dose of the TRH analog RX 77368 elicited modest effects in normoglycemic rats of both sexes, whereas in Goto–Kakizaki rats, a genetic model of type 2 diabetes, the same dose induced marked sex-dependent responses, including hyperglycemia with pronounced hyperinsulinemia in males and a glucose-lowering effect accompanied by moderate insulinotropic activity in females. Similarly, Bacová et al. (2005) demonstrated that neonatal TRH treatment in streptozotocin-exposed rats induced long-term, sex-dependent alterations in pancreatic endocrine function, reflected by differences in glycemic control *in vivo* and insulin secretory responses to glucose *in vitro*. Together, these findings support the concept that TRH signaling modulates metabolic regulation in a sexually dimorphic manner.

Given the established role of the HPT axis in osmoregulatory adaptation in teleost fish, particularly through its functional integration with cortisol, GH, and prolactin (Deal and Volkoff, 2020), it is pertinent to consider whether the metabolic effects observed in glucose-immersion models could be confounded by osmotic stress. Although the direct involvement of TRH in osmoregulation remains poorly defined in teleosts, TRH is known to modulate several neuroendocrine axes, including GH, prolactin, and the hypothalamus–pituitary–interrenal (HPI) axis, all of which participate in hydromineral homeostasis (Lazcano et al., 2025; Rotllant et al., 2000). Because glucose immersion inevitably increases environmental osmolarity, nonspecific stress-related effects cannot be entirely excluded *a priori*.

Nevertheless, available experimental evidence strongly supports that hyperglycemia induced by glucose immersion is predominantly metabolic rather than osmotic in origin. Using the same hyperglycemia model applied in the present study, McCarthy et al. (2021) demonstrated that progressive glucose exposure (55–166 mM) increased blood glucose levels, whereas an osmotic control using mannitol did not, indicating that hyperglycemia depends on active glucose uptake and metabolism. Similarly, Tanvir et al. (2018) reported that exposure to 111 mM glucose induced hyperglycemia and associated physiological alterations that were not reproduced by mannitol treatment, despite comparable osmotic load. These findings indicate that glucose-immersion models effectively dissociate metabolic from osmotic effects.

Consistent with this interpretation, estimation of the osmotic contribution of the 111 mM glucose treatment employed here indicates that environmental osmolarity (∼115–125 mOsm/kg) remains markedly lower than zebrafish plasma osmolarity (∼280–330 mOsm/kg; Lawrence, 2007; Kozłowski et al., 2018), maintaining a strongly hypotonic external milieu. Together, experimental evidence and osmolarity estimates support that the hyperglycemic phenotype observed in this model is largely driven by metabolic mechanisms.

Nonetheless, given the sensitivity of TRH signaling to metabolic and stress-related cues, a contributory role of osmotic factors cannot be fully excluded and warrants cautious interpretation. In this context, the absence of a hypoglycemic response to TRH in females may reflect sex-dependent interactions between metabolic state and osmotic or stress-related signals. Moreover, intrinsic sex-dependent differences in the expression of TRHergic components identified in this study, particularly the increased *trh-de* expression in peripheral tissues, may further limit TRH bioavailability and systemic efficacy in females.

### Transcriptional regulation associated with TRH-mediated metabolic modulation

At the molecular level, our findings indicate that TRH contributes to glucose homeostasis in zebrafish through mechanisms that extend beyond a direct insulinotropic effect. Although hyperglycemia elicited a compensatory increase in pancreatic *insa* expression in both sexes, as previously described in this model (Nipu et al., 2022), TRH treatment reduced circulating glucose levels without enhancing insulin gene expression, particularly in males, which exhibited a more favorable metabolic profile. Collectively, these observations suggest that the hypoglycemic effect of TRH in zebrafish is mediated by insulin-independent mechanisms, potentially involving central autonomic regulation, modulation of hepatic glucose production, and/or counterregulatory hormone signaling (Fekete and Lechan, 2014; Joseph-Bravo et al., 2015), thereby reducing the need for sustained insulin signaling.

In this context, the absence of an evident insulinotropic effect in our study does not exclude a role for TRH in insulin secretion but rather suggests that such effects may depend on the temporal window of evaluation. In mammalian models, TRH has been shown to transiently modulate insulin secretion (Najvirtová et al., 2005; Štrbák, 2018); however, in our experimental design, analyses were performed 3 days after treatment. Therefore, early and short-lived effects on insulin expression or secretion may not have been captured.

At the pancreatic level, TRH administration modulated the transcriptional regulation of *trh* and *trh-de* in a sex-dependent manner, suggesting the activation of endogenous regulatory mechanisms under hyperglycemic conditions. Previous studies support the capacity of TRH to regulate components of its own signaling pathway (Cook and Hinkle, 2004; Harris et al., 2001; Heuer et al., 1998; Pech-Pool et al., 2020). In this regard, our pancreatic data support the notion that TRH functions as a modulator that adjusts pancreatic responses according to metabolic demand.

In the liver, our data further support the idea that TRH does not act as an insulinotropic factor but instead functions as an endocrine–metabolic modulator influencing glycemic regulation through hepatic mechanisms. These may include the regulation of hepatic glucose production, tissue insulin sensitivity, the balance between anabolic and catabolic pathways, and mitochondrial activity (Rocha et al., 2015; Yang et al., 2018), all of which warrant further detailed investigation. The observation that hyperglycemia reduced hepatic *trh* expression in males suggests that, under conditions of elevated energetic demand, the liver attenuates its local TRHergic tone as a homeostatic mechanism. This reduction may limit excessive activation of metabolic pathways associated with cellular stress, lipogenesis, or energetic imbalance, which have been described in zebrafish in the context of sustained neuroendocrine axis activation (Faught and Vijayan, 2019; Nipu et al., 2022; Ruiz-Jarabo et al., 2018). The further decrease in hepatic *trh* expression following TRH treatment reinforces the presence of tissue-level homeostatic regulation. Notably, the upregulation of the *trh-r2* receptor in the female liver suggests the activation of sex-dependent TRHergic pathways involved in metabolic–endocrine regulation, which merit further investigation.

Taken together, these findings indicate that TRH act as a modulatory hormone with fine-tunes metabolic responses and could contribute to the preservation of cellular homeostasis under hyperglycemic conditions. Moreover, our results situate zebrafish as a valuable model for investigating sex-dependent metabolic actions of TRH at both central and peripheral levels.

## MATERIALS AND METHODS

### Zebrafish husbandry

Adult zebrafish (*Danio rerio*) of both sexes were obtained from a commercial aquarium and kept under controlled laboratory conditions in a recirculating water system. Environmental parameters were maintained as follows: a 10 h light/14 h dark photoperiod, water temperature at 28±1°C, pH between 7.4 and 7.8, and conductivity ranging from 500 to 600 µS/cm. Fish were fed twice daily with a mixed diet of live Artemia salina (brine shrimp) and commercial flake food containing 42% protein, 5% fat, 6% fiber, 11% ash, and 8% moisture (Biomaa), in accordance with the zebrafish husbandry guidelines by Westerfield (Westerfield, 2000) and the Zebrafish Information Network (ZFIN; https://zfin.org/).

All experimental procedures were approved by the Bioethics Committee of the Instituto de Neurobiología, Universidad Nacional Autónoma de México (UNAM), and the Universidad Autónoma de Querétaro (UAQ) under protocols #152.A and #13830, respectively. All activities were conducted in accordance with the International Guidelines for the Care and Use of Animals in Biomedical Research (Jones-Bolin, 2012).

### Nomenclature

Gene and protein abbreviations were assigned following the Zebrafish Nomenclature Conventions: zebrafish genes and transcripts were denoted in lowercase italics (‘*aaa*’) and their proteins in regular font with an initial capital letter (‘Aaa’). Mammalian genes were indicated in uppercase italics (‘*AAA*’) and mammalian proteins in uppercase regular font (‘AAA’).

### Bioinformatic analysis and protein modeling

The amino acid sequences of zebrafish TRH, TRH-Rs, and TRH-DE were retrieved from the Ensembl (https://www.ensembl.org) and NCBI (https://www.ncbi.nlm.nih.gov) databases. Sequence identity and annotation accuracy were verified by reciprocal BLAST searches and multiple sequence alignments performed with Clustal Omega (EMBL-EBI) and visualized using ESPript 3.0. Representative molecular models of each protein were constructed with IBS Illustrator for Biological Sequences to depict conserved domains and key functional residues. Detailed sequence alignments and phylogenetic comparisons with representative vertebrate species are provided in Figs S1–S4 and Table S1.

### Zebrafish embryos and larval sampling

Embryos were obtained after controlled mating of adult breeders placed in breeding chambers equipped with mesh grids to prevent egg predation. Embryos at different cell stages were collected in accordance with the criteria established by Kimmel et al. (1995). Newly fertilized embryos were considered at 0 days post-fertilization (0 dpf) and subsequently collected at the following developmental stages: 0.5 dpf (12 hpf), 1 dpf, 2 dpf, 3 dpf, 4 dpf, and 5 dpf (starting from 3 dpf, organisms were classified as larvae). All embryos were incubated at 28±1°C in E3 medium (5 mM NaCl, 0.17 mM KCl, 0.33 mM CaCl₂, 0.33 mM MgSO₄, and Methylene Blue).

For molecular analyses, each biological replicate consisted of a single pool of ten embryos or larvae, as described in detail in the ‘RNA isolation and cDNA synthesis’ section for gene expression assays. Samples were carefully collected using wide-bore pipette tips to minimize mechanical damage (Vergauwen et al., 2018). Specimens were euthanized by immersion in pre-chilled E3 medium at 4°C, a procedure that induces rapid hypothermic anesthesia followed by loss of vital functions, in accordance with accepted guidelines for zebrafish larval euthanasia (ZFIN). Immediately after euthanasia, samples were preserved in Trizol reagent (Invitrogen, Carlsbad, CA, USA) and stored at −80°C until further processing.

### Adult zebrafish tissue sampling

Anesthesia and euthanasia were conducted using the hypothermic shock method (Eames et al., 2010) to minimize potential alterations in blood glucose levels. Adult zebrafish (>3 months to 1 year of age) were rapidly immersed in water at 12°C for anesthesia and at 4°C for euthanasia.

Sex was initially determined based on external secondary sexual characteristics, such as yellow pigmentation on the anal fin and ventral region in males and abdominal distension in females (Robison et al., 2008). The sex was subsequently confirmed through postmortem gonadal inspection.

For characterization analyses, tissue dissections were performed immediately after euthanasia to collect samples from the brain (from cerebellum to telencephalon), retina, heart, liver, pancreas, muscle, skin, and the whole GI tract, encompassing both CNS and peripheral tissues, as detailed in the ‘RNA isolation and cDNA synthesis’ section for gene expression assays. All samples were preserved in Trizol reagent and stored at −80°C until further molecular analysis.

### Dissection and validation of pancreatic tissue of zebrafish

Given the small size and anatomically dispersed nature of the adult zebrafish pancreas, which makes its isolation technically challenging (Houbrechts et al., 2019; Maddison et al., 2015), pancreatic tissue was dissected using an anatomically guided approach based on established histological landmarks (Chen et al., 2007; Menke et al., 2011; Pérez Díaz et al., 2025). Spatial orientation was determined from serial histological sections (10 µm) of whole adult zebrafish, allowing identification of exocrine acinar clusters and principal endocrine islets. Guided by these references, fresh dissections were performed under a stereomicroscope (Zeiss Discovery V12), and the putative pancreatic tissue was identified and excised as a single unit.

Tissue identity was confirmed by histological analysis of pancreatic architecture using Hematoxylin and Eosin staining, immunofluorescent detection of insulin-positive endocrine cells using a zebrafish-specific anti-insulin primary antibody (rabbit anti-insulin, Abcam, #ab210560; 1:500) and an Alexa Fluor 488-conjugated anti-rabbit IgG secondary antibody (Invitrogen), and PCR amplification of pancreas-specific endocrine markers (insulin a; *insa* and glucagon b; *gcgb*) from reverse-transcribed RNA. Representative histological, immunofluorescence, and molecular validation images are provided in Figs S5–S7.

### RNA isolation and cDNA synthesis

Total RNA was isolated using Trizol reagent according to the manufacturer's instructions. For embryo/larval stages, total RNA was extracted from pools of individuals defined for each biological replicate and developmental stage. For adult samples, tissues were processed individually, as described in detail in subsequent sections.

RNA purity and concentration were assessed using a NanoDrop Lite Plus spectrophotometer (Thermo Fisher Scientific, Waltham, MA, USA), and RNA integrity was confirmed by 2% agarose gel electrophoresis.

For cDNA synthesis, 1 µg of total RNA was used as a template for all samples with the RevertAid First Strand cDNA Synthesis Kit (Thermo Fisher Scientific, Cat. No. K1641, V. A. Graiciuno 8, Vilnius, LT-02241 Lithuania), according to the manufacturer's protocol.

### Quantitative Real-Time PCR (qPCR)

qPCR was performed using a QuantStudio 1 Real-Time PCR System (Applied Biosystems, Thermo Fisher Scientific, Waltham, MA, USA) and SYBR Green-based detection. Reactions were carried out in a final volume of 8 µl, comprising 3.3 µl of Maxima SYBR Green/ROX qPCR Master Mix 2X (Thermo Fisher Scientific, Cat. No. K0221, Vilnius, Lithuania), 0.32 µl of each primer (10 µM), and 2 µl of cDNA. For embryo/larval samples, cDNA was diluted in a 1:5 ratio and adjusted to the final volume with nuclease-free water. For adult samples, undiluted cDNA was used.

PCR amplification conditions were as follows: 95°C for 10 min, followed by 40 cycles at 95°C for 15 s and 60°C for 1 min. A melting curve analysis was performed at the end of each run to assess amplification specificity. All reactions were conducted in technical duplicates. Primer specificity and amplification efficiency were validated by standard curve analysis using serial dilutions of cDNA prior to the experimental runs (Table S2).

For all gene expression assays, *n*=5 biological replicates per condition were analyzed, with each sample measured in technical duplicate. In developmental analyses, whole embryos/larvae were pooled from ten individuals per biological replicate. In adult zebrafish, brain, retina, liver, muscle, skin, and GI tissues were collected and processed individually, with each biological replicate corresponding to one animal. Due to tissue size limitations, pancreatic and heart samples were pooled from three animals per biological replicate, resulting in five independent pools per condition. This sampling strategy ensured sufficient RNA yield while maintaining biological replication, consistent with previous zebrafish gene expression studies (Mills and Gallagher, 2017; Råbergh et al., 2000). Sample size was determined based on prior zebrafish gene expression studies demonstrating reliable detection of biologically relevant effect sizes with *n*=5. This number was considered sufficient to detect statistically meaningful differences. No formal *a priori* power calculation was performed.

For embryonic and larval stages, *18S* rRNA and *lsm12b* were used as reference genes, as recommended for zebrafish embryonic and larval development due to their stable expression across ontogenetic stages (Hu et al., 2016), with 0 dpf as the calibrator. In adult tissues, gene expression was normalized against *actb1* (*β-actin*) and *18S* rRNA, using brain tissue as the reference sample. The relative content of all mRNA transcripts was determined by the comparative threshold cycle (Ct) method using the formula 2^^−ΔΔCt^, in which expression levels were normalized to the geometric mean of the corresponding reference genes.

Primer sequences used in this study are provided in Table S3.

### Hyperglycemia induction and *in vivo* TRH treatment

Adult zebrafish (>3 months to 1 year old) underwent a 2-week acclimatization period under controlled laboratory conditions prior to experimentation. Throughout acclimatization and the subsequent treatment phase, fish were gently handled to minimize stress and fed twice daily. Following acclimatization, a total of 45 adults were randomly assigned to three experimental groups (*n*=15/group): (1) a control group maintained in glucose-free water and receiving a daily intraperitoneal (ip) injection of vehicle (0.9% saline), (2) a glucose group exposed to 111 mM D-glucose and daily injected with vehicle, and (3) a glucose+TRH group exposed to the same glucose concentration but receiving ip TRH [1 µg per gram body weight (BW)] injections. Experiments were conducted independently for males and females to evaluate sex-specific responses.

Hyperglycemia was induced according to the model described by Capiotti et al. (2014), in which continuous immersion in 111 mM D-glucose for 3 days produces a sustained hyperglycemic state in adult zebrafish. Given that glucose immersion inevitably increases environmental osmolarity, and considering the well-established involvement of the HPT axis in osmoregulatory processes in teleost fish (Deal and Volkoff, 2020), potential osmotic contributions were taken into account. However, previous studies have demonstrated that hyperglycemia induced by glucose immersion does not result from osmotic disturbances capable of altering circulating glucose levels. Specifically, plasma glucose concentrations in osmotic control groups remain comparable to those of untreated fish (McCarthy et al., 2021; Tanvir et al., 2018). A detailed discussion of osmotic versus metabolic contributions in this model is provided in the Discussion section.

The glucose solution was prepared from a 50% stock (PiSA Farmacéutica^®^, Guadalajara, Jalisco, Mexico). TRH (Bachem, Cat. No. 4038214, Bubendorf, Switzerland) was administered intraperitoneally at a dose of 1 µg/g BW, delivered in an injection volume of 1 µl/g BW. Based on body mass, the approximate injection volume ranged from 0.7 to 1.0 µl per fish, with a mean body mass of 0.779±0.148 g for males and 0.897±0.174 g for females. Prior to selecting this dose, a dose–response assay was performed evaluating 0.1, 1, and 10 µg TRH/g BW, based on previous studies conducted in other teleost species and in rodent models (Abbott and Volkoff, 2011; Luo et al., 2008). The 1 µg/g dose was chosen as it produced a consistent physiological response without signs of stress or adverse effects (Fig. S8).

All injections were performed once daily at approximately 09:00 to minimize circadian variability, following the procedure described by Kinkel et al. (2010). Vehicle injections were administered in equivalent volumes to maintain procedural consistency across groups.

The experimental sequence, summarized in Fig. S9, consisted of three consecutive days of TRH or vehicle administration concurrent with glucose exposure, followed by sampling on day 3. Briefly, on day 0, after randomization, fish received the first ip injection, initiating both the dosing regimen and glucose immersion. Injections were repeated on days 1 and 2 at the same time each morning. Following the final injection and morning feeding on day 2, food was withdrawn to establish a 24 h fasting period prior to sampling. No injections were performed on day 3. On the morning of day 3, fish were transferred to glucose-free water for 15 min before euthanasia and sampling. Blood was then collected, and liver was dissected and stored individually, while pancreatic tissue was pooled from three fish per sample to ensure sufficient RNA yield for molecular analyses. Pancreatic samples were used to quantify the expression of *insa*, the insulin isoform functionally relevant in adult zebrafish (Mullapudi et al., 2018), together with key components of the TRHergic system (*trh*, *trh-r1a*, and *trh-de*). Hepatic samples were analyzed to assess the expression of glucose transporters, including *slc2a2* (GLUT2) and *slc2a12* (GLUT12) (Houbrechts et al., 2019), as well as the insulin receptor isoforms *ins-ra* and *ins-rb*, and TRHergic signaling elements (*trh*, *trh-r1a*, *trh-r2*, *trh-r3*, and *trh-de*). All tissues were immediately placed in Trizol reagent and stored at −80°C until processing.

To validate the responsiveness of the hyperglycemia model, an independent experiment was conducted using insulin as a positive control. Adult male zebrafish were subjected to the same glucose-immersion protocol described above (Fig. S9). A total of 12 fish were distributed into a glucose+vehicle group and a glucose+insulin group (*n*=6 per group). Regular human insulin (100 IU/ml injectable solution; AMSA^®^, Mexico City, Mexico) was used.

Fish received a single ip injection of insulin at a dose of 1 IU/kg BW, administered in a volume of 1 µl/g BW, following a protocol previously described (Capiotti et al., 2014). The insulin stock solution (100 IU/ml) was freshly diluted 1:100 in sterile 0.9% saline to obtain a working concentration of 1 IU/ml (0.001 IU/µl), adjusted to the individual BW of each fish. Prior to glucose determination, fish were transferred to glucose-free water for 15 min to prevent environmental glucose contamination. Blood glucose levels were quantified 1 h after insulin administration. The resulting data are presented in Fig. S10.

### Glucose measurement

Blood glucose levels were determined immediately after collection by caudal transection, following the 24 h fasting period described above. To prevent contamination from residual external glucose, fish were first transferred to glucose-free water for 15 min prior to sampling. Glucose concentrations were measured using a portable glucometer (Accu-Chek, Roche Diabetes Care, Mexico City, Mexico).

### Statistical analysis

All statistical analyses and graphical representations were performed using GraphPad Prism version 10 (GraphPad Software, Boston, MA, USA).

Data are presented as mean±s.e.m. Data distribution was assessed for normality using the Shapiro–Wilk test prior to parametric analysis, and homogeneity of variance was evaluated when appropriate. Depending on the comparison, groups were analyzed using Student's *t*-test or one-way ANOVA followed by Tukey's post hoc test. *P*<0.05 was considered statistically significant.

No animals or samples were excluded from the analysis, and no outliers were removed. Investigators were aware of group allocation during treatment administration and outcome assessment.

## Supplementary Material

10.1242/biolopen.062418_sup1 Supplementary information
