# Supplementary material for: Developmental and tissue-specific expression of thyrotropin-releasing hormone signaling genes in zebrafish and its association with glycemic regulation
Source: Biol Open. 2026 Apr 2;15(3):bio062418. doi: 10.1242/bio.062418 (PMC13072134; doi:10.1242/bio.062418)
Supplement: Supplementary information [file biolopen-15-062418-s1.pdf]

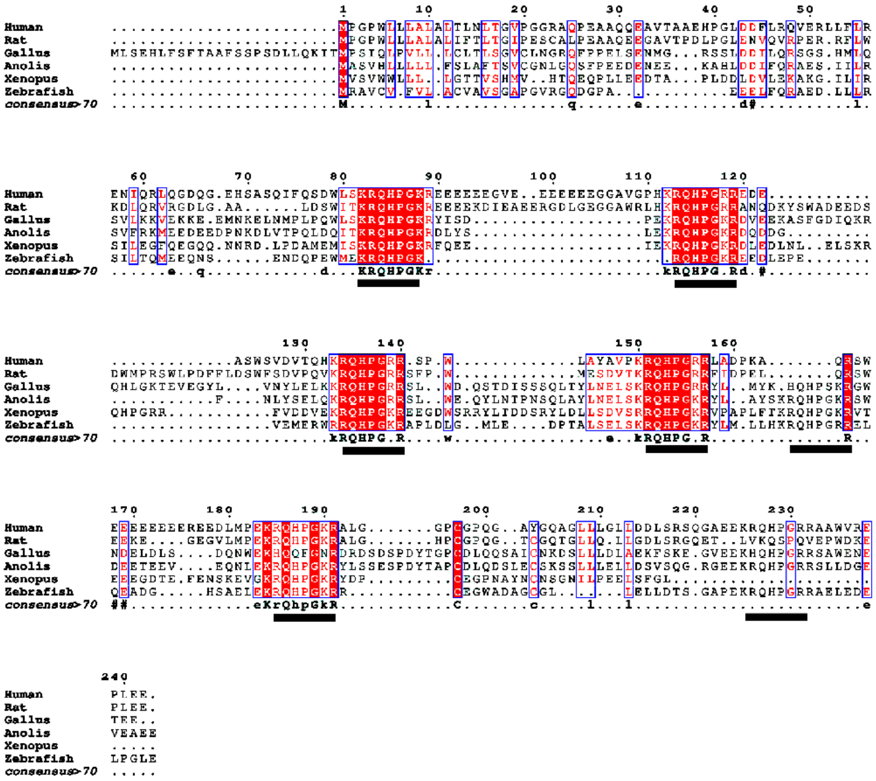

Fig. S1. Comparative alignment of pre proTRH amino acid sequences across representative vertebrate species. A multiple sequence alignment of the deduced amino acid sequences of the prepro-TRH was generated using ESPript 3.0. Representative vertebrate species include mammals (*Homo sapiens*, *Rattus norvegicus*), birds (*Gallus gallus*), reptiles (*Anolis carolinensis*), amphibians (*Xenopus tropicalis*), and fish (*Danio rerio*). Identical residues are highlighted in red, and similar residues with chemical similarity are boxed in blue. Dots (·) denote residues identical to the consensus sequence, whereas dashes (–) indicate non-similar residues. The conserved QHPG motifs corresponding to the TRH progenitor sequences are evident throughout the alignment, and the flanking basic residues lysine (K) and arginine (R) represent potential cleavage sites for the generation of mature TRH peptides. Black bars below the alignment indicate the positions of the proTRH motifs identified in *Danio rerio*. Consensus residues with ≥70% identity across species are shown below the alignment.

Biology Open • Supplementary information

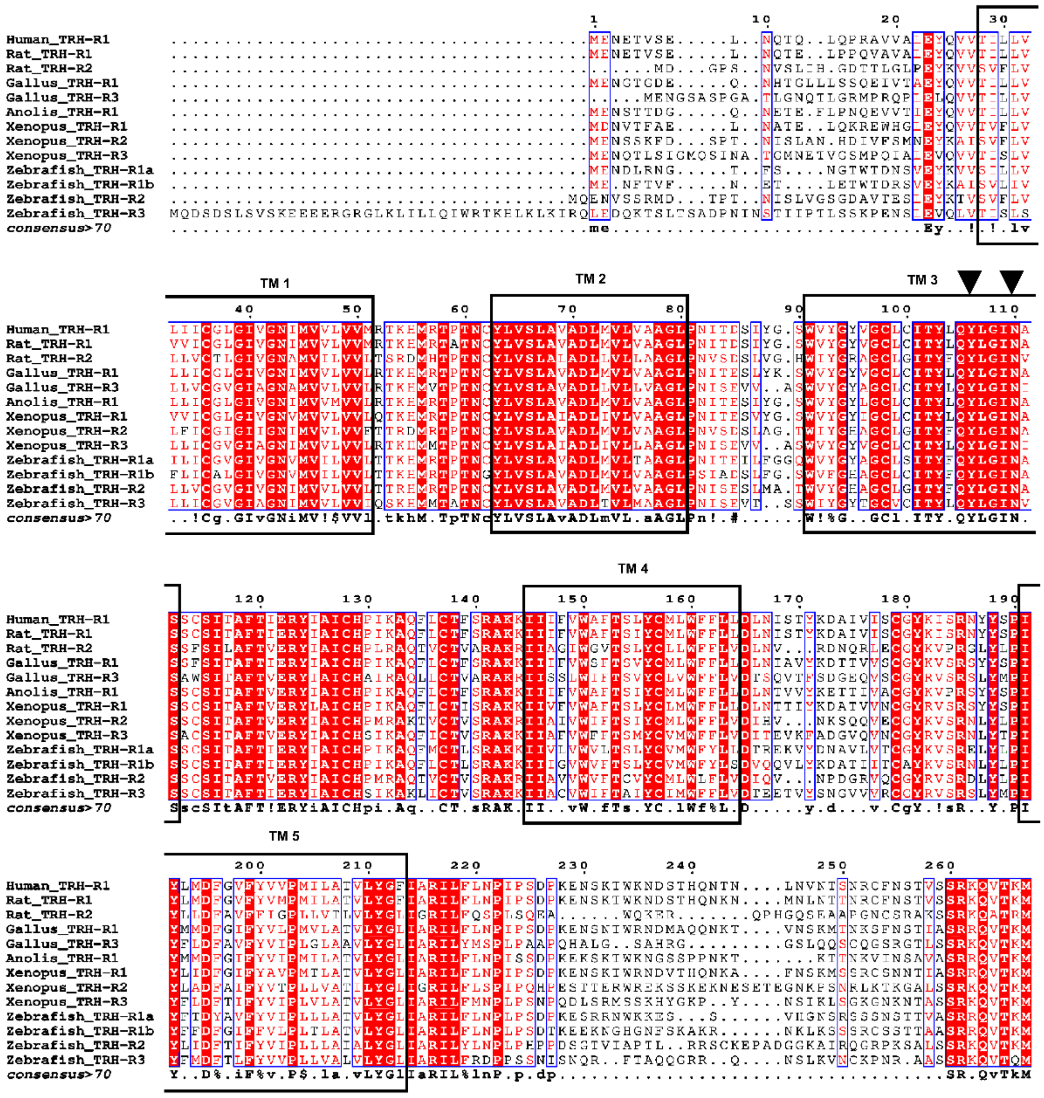

Biology Open • Supplementary information

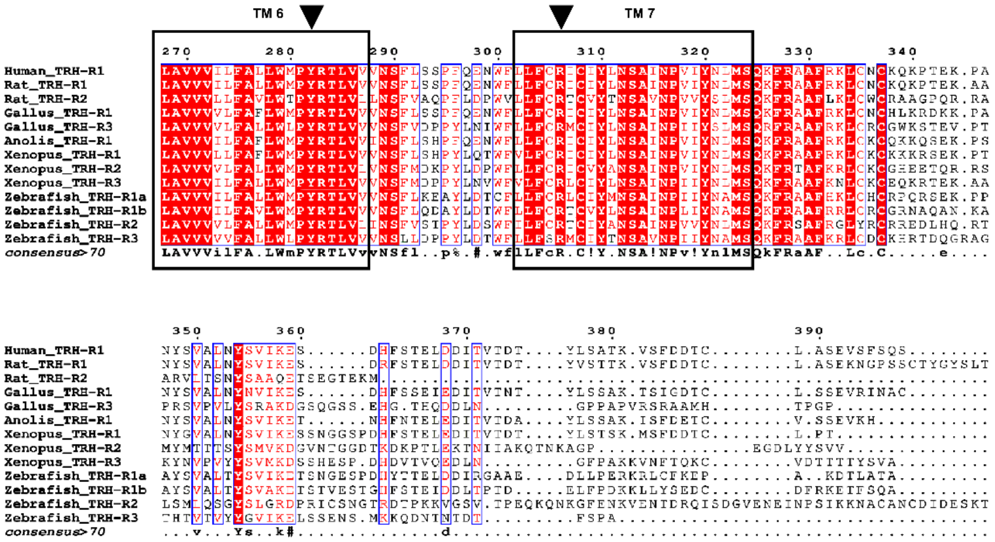

Fig. S2. Comparative alignment of TRH-Rs isoforms across representative vertebrate species. A multiple sequence alignment of the deduced amino acid sequences of TRH-R isoforms was generated using ESPript 3.0. Representative species include mammals (*Homo sapiens*, *Rattus norvegicus*), birds (*Gallus gallus*), reptiles (*Anolis carolinensis*), amphibians (*Xenopus tropicalis*), and fish (*Danio rerio*). Identical residues are shown in red, and chemically similar residues are boxed in blue. Dots (·) indicate residues identical to the consensus sequence, while dashes (–) denote non-similar residues. Predicted transmembrane domains (TM1–TM7), defined according to the human TRH-R reference sequence, are indicated by black boxes. Black arrowheads mark conserved residues proposed to participate in TRH binding, including tyrosine (Tyr) and asparagine (Asn) in TM3, tyrosine (Tyr) in TM6, and arginine (Arg) in TM7. Consensus residues with ≥70% identity across species are shown below the alignment. Residue numbering follows that of the human reference sequence.

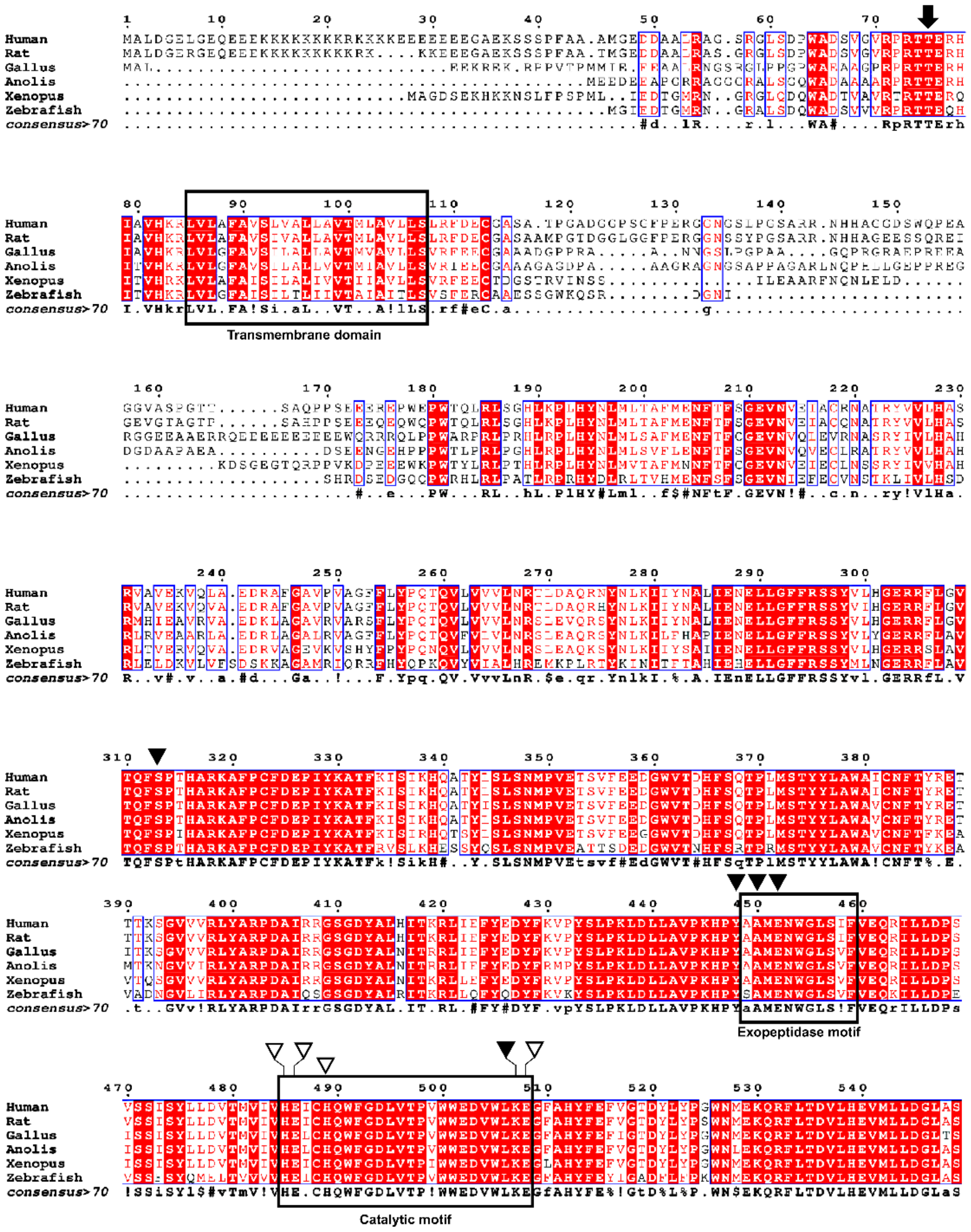

Biology Open • Supplementary information

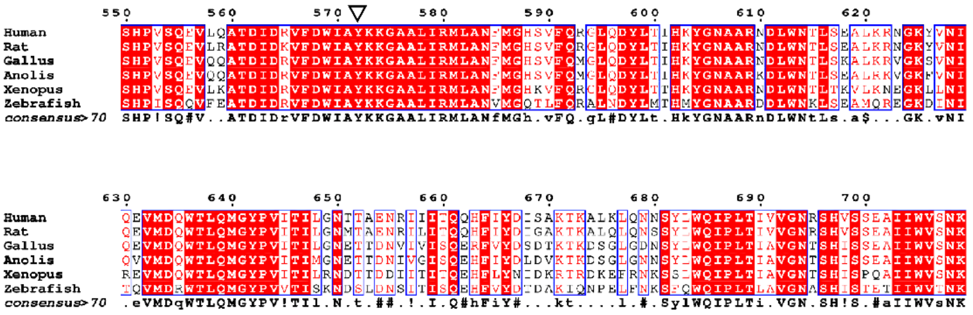

Fig. S3. Comparative alignment of putative TRH-DE sequences across representative vertebrates. A multiple sequence alignment of the deduced amino acid sequences of TRH-DE orthologs was generated using ESPript 3.0. Representative vertebrate species include mammals (*Homo sapiens*, *Rattus norvegicus*), birds (*Gallus gallus*), reptiles (*Anolis carolinensis*), amphibians (*Xenopus tropicalis*), and fish (*Danio rerio*). Identical residues are highlighted in red, and chemically similar residues are boxed in blue. Dots (·) indicate residues identical to the human reference sequence, whereas dashes (–) denote gaps or non-conserved positions. The alignment highlights the canonical motifs of M1 zinc-dependent metallopeptidases, including the transmembrane domain, AAMEN exopeptidase motif, and HEXXHX$_{18}$E catalytic motif (black boxes). Black arrowheads mark residues proposed to participate in TRH substrate recognition (*Danio rerio*: Ser227, Tyr361, Ala363, Glu365, and Lys421), while white arrowheads denote catalytically essential residues involved in peptide bond hydrolysis (*Danio rerio*: His399, Glu400, His403, Glu422, and Tyr486). A black arrow indicates a predicted intracellular phosphorylation site (*Danio rerio*: Thr29), potentially regulated by protein kinase C. Residue numbering follows that of the human reference sequence.

Biology Open • Supplementary information

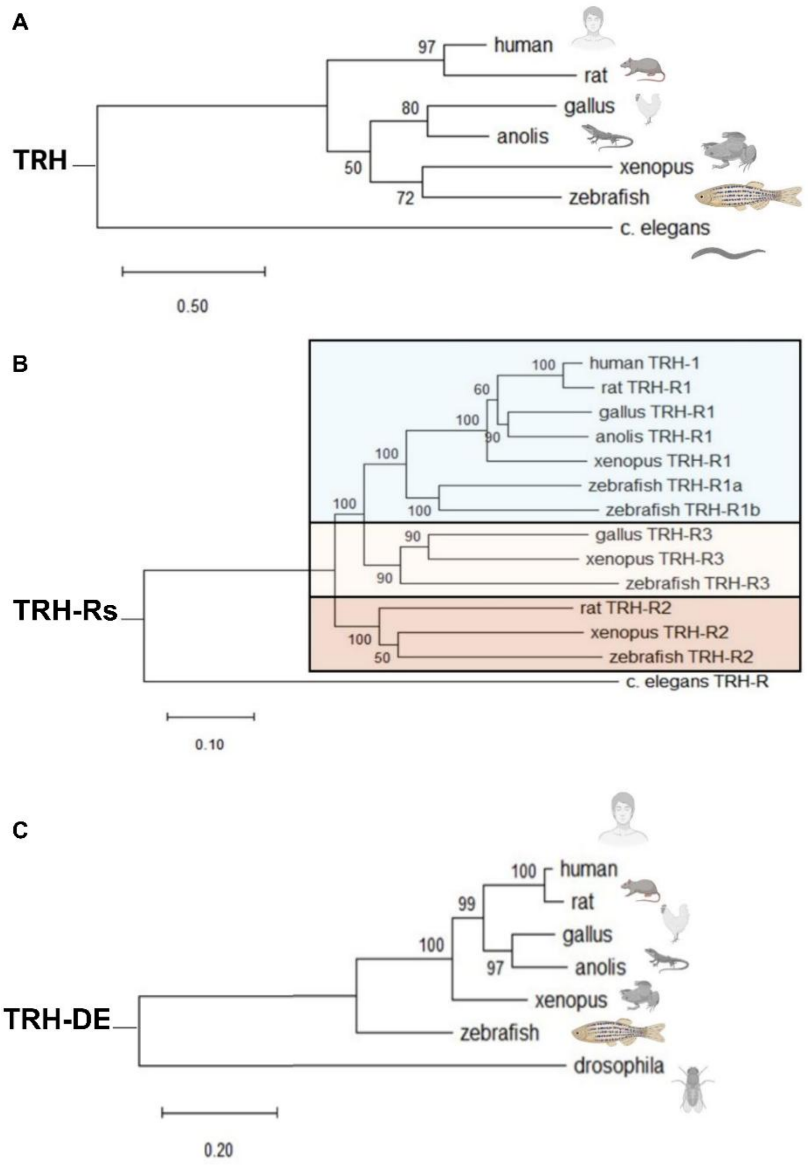

Fig. S4. Phylogenetic analysis of TRH, TRH-Rs, and TRH-DE amino acid sequences. Phylogenetic trees are shown for **(A)** TRH, **(B)** its receptors (TRH-Rs), and **(C)** the TRH-degrading enzyme (TRH-DE). Evolutionary relationships were inferred using the Neighbor-Joining method (Saitou and Nei, 1987). The optimal trees, with a total branch length of 3.220, are shown. Numbers next to branches indicate the percentage of replicate trees in which the associated taxa clustered together in the bootstrap test (1,000 replicates; Felsenstein, 1985). Trees are drawn to scale, with branch lengths proportional to evolutionary distances calculated using the Poisson correction method (Zuckerkandl and Pauling, 1965), expressed as the number of amino acid substitutions per site.

The analysis included fourteen amino acid sequences, and ambiguous positions were handled using the pairwise deletion option, resulting in a final dataset of 562 positions. Phylogenetic analyses were performed in MEGA X (version 12). Illustrations of representative species were created with BioRender.com (agreement number: AF28PRKRQR).

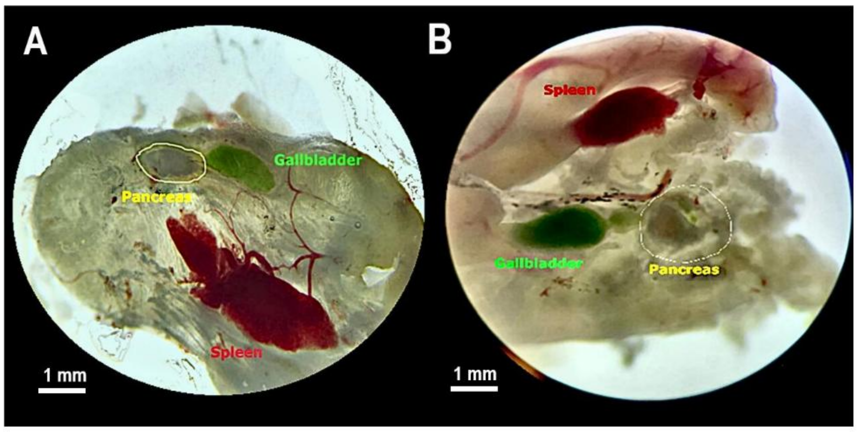

Fig. S5. Approximate spatial localization of pancreatic tissue in adult zebrafish. (A, B) Identification of major digestive organs, including the spleen (red), gallbladder (green), pancreas (yellow), and gastrointestinal tract. Specimens were immersed in PBS and examined under a stereomicroscope equipped with a digital camera using incident light. Structures were digitally annotated to facilitate anatomical recognition. Scale bar: 1 mm.

Biology Open • Supplementary information

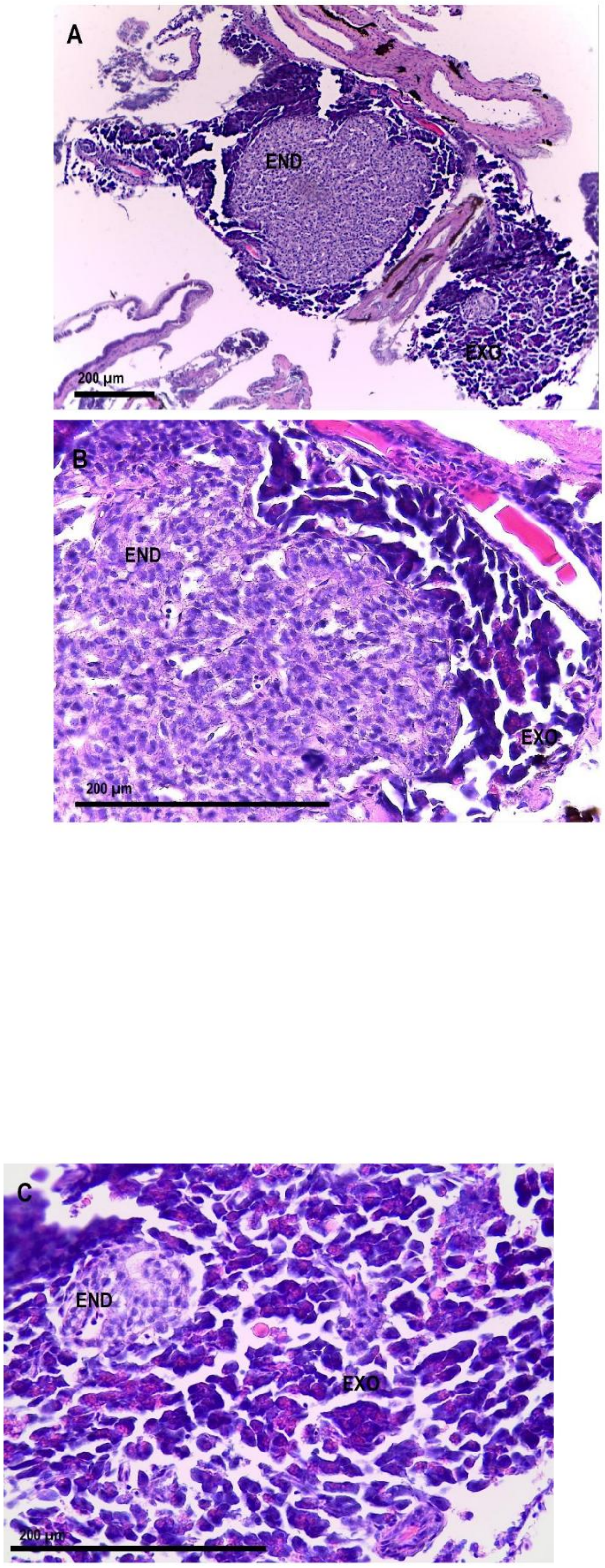

Fig. S6. Identification of pancreatic tissue in histological sections of adult zebrafish stained with hematoxylin and eosin. Panels **A–C** show different magnifications and views of pancreatic islets (END) surrounded by exocrine acinar tissue (EXO).Sections were cut at 10 µm thickness and imaged with a light microscope. Structures were digitally annotated for clarity. Scale bars: 200 µm.

Biology Open • Supplementary information

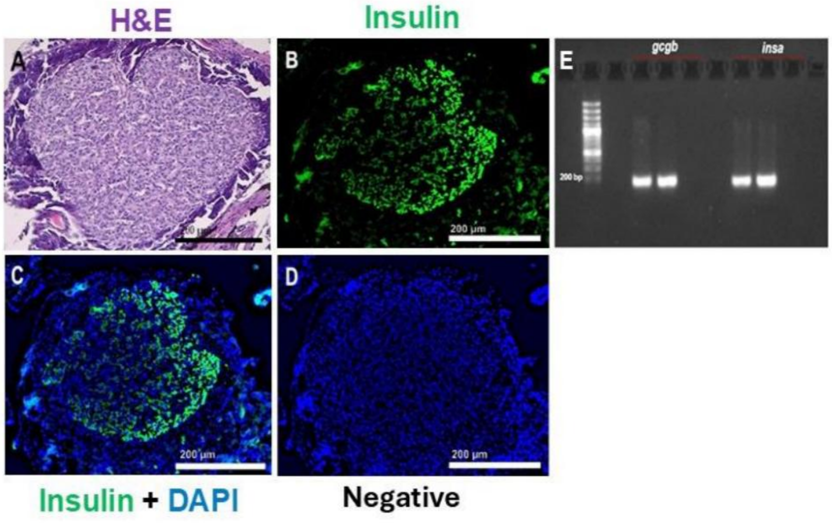

Fig. S7. Detection of insulin in adult zebrafish pancreatic tissue by immunofluorescence and analysis of *gcgb* and *insa* transcripts by RT-PCR. Panels **A–D** show 10 µm paraffin sections of adult zebrafish pancreas. **(A)** Hematoxylin and eosin staining of an endocrine pancreatic islet. **(B)** Immunofluorescent detection of insulin was performed using a rabbit polyclonal anti-zebrafish insulin primary antibody (Abcam, Cambridge, UK; catalog no. ab210560) at a working dilution of 1:500. After incubation with the primary antibody, sections were washed three times in PBS. The primary antibody was detected using an Alexa Fluor 488–conjugated goat anti-rabbit IgG secondary antibody (Invitrogen, Waltham, MA, USA; catalog no. A-11008) at a dilution of 1:1000, followed by three additional PBS washes. **(C)** Merged image including DAPI nuclear counterstain. **(D)** Negative control processed in parallel without primary antibody. Scale bars: 200 µm.

The anti-insulin antibody has been validated for use in zebrafish tissue; specificity is supported by the absence of signal in the no-primary control (panel D) and is consistent with the supplier's validation profile for ab210560 (Abcam). This antibody has also been previously used in zebrafish pancreatic tissue in the study by Faraj et al. (2025), *Pancreatic exocrine damage induces beta cell stress in zebrafish larvae*, Diabetologia, 68(8), 1754–1768. https://doi.org/10.1007/s00125-025-06432-4.

(E) Agarose gel electrophoresis showing PCR amplification of pancreatic endocrine markers glucagon b (*gcgb*) and insulin a (*insa*) from reverse-transcribed RNA. Primer sequences: insa forward 5′-TAAGCACTAACCCAGGCACA-3′ and reverse 5′-TCCTGGGCAGATTTAGGAGGA-3′; gcgb forward 5′-GACCAGGAGAGCACAAGACT-3′ and reverse 5′-CGTCGTCCTCGTCCTGTTTT-3′.

Biology Open • Supplementary information

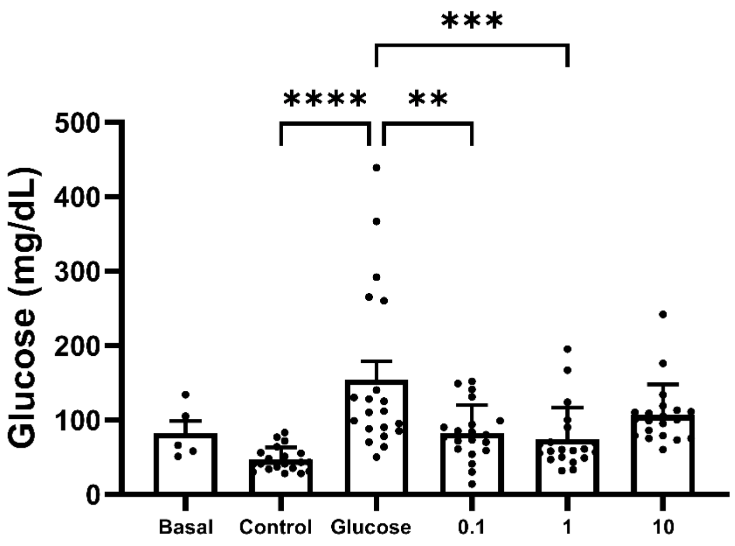

Fig. S8. Dose–response assay of TRH on blood glucose levels in adult zebrafish. Basal: fish maintained under normal feeding conditions without fasting or treatment; Control: fasted fish injected with vehicle (sham) without glucose exposure; Glucose: fish exposed to 111 mM D-glucose and injected with vehicle; 0.1, 1, 10: fish exposed to 111 mM D-glucose and injected intraperitoneally with 0.1, 1, or 10 µg TRH per gram of body weight, respectively. All treatments were performed for three consecutive days following the protocol described in Section 2.9. Each group contained n = 20 fish. Data from two independent experiments were pooled and are presented as mean ± SEM. Statistical analysis was performed using one-way ANOVA followed by Tukey's multiple comparisons test (two-tailed). Statistical significance is indicated as follows: ***p < 0.01; ***p < 0.001; ****p < 0.0001.*

Biology Open • Supplementary information

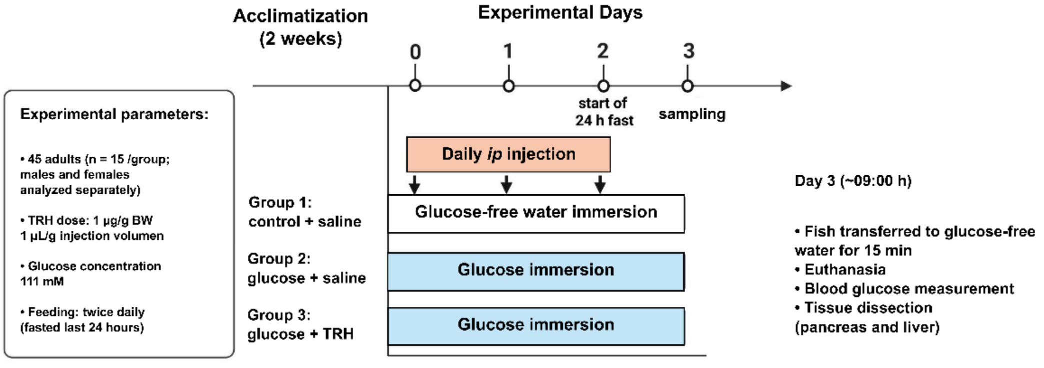

Fig. S9. Schematic representation of the *in vivo* experimental procedure. The diagram summarizes the sequential phases of the experiment, including the acclimation period, the induction of hyperglycemia by glucose immersion, the intraperitoneal (*ip*) TRH injection schedule, the fasting period, and the subsequent sampling procedures in adult zebrafish. All experimental procedures were conducted in both sexes and analyzed separately. Figure created with BioRender.com (Agreement number: PD291I3XQ9).

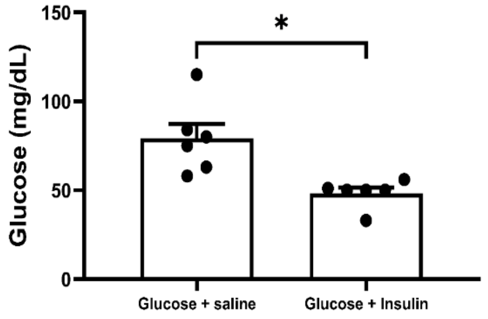

Fig. S10. Insulin administration reduces blood glucose levels in hyperglycemic adult male zebrafish. Adult male zebrafish were exposed to glucose immersion and subsequently treated with either vehicle (saline) or human insulin (1 IU/kg body weight; n = 6 per group). Insulin was administered as a single intraperitoneal injection at a volume of 1 µL/g BW. Blood glucose levels were measured 1 h after injection. Data are shown as individual values with bars representing the mean ± SEM. Statistical analysis was performed using an unpaired two-tailed Student's *t*-test. Statistical significance was observed between groups (*$*p < 0.05$*). Data are representative of one independent experiment.

Biology Open • Supplementary information

Table S1. Accession numbers of protein sequences corresponding to vertebrate TRHergic system components

Species / Protein	Prepro-TRH	TRH-Rs			TRH-DE
		TRH-R1	TRH-R2	TRH-R3	
Homo sapiens	ENSP00000303452	NP_003292.1	N/A	N/A	ENSP00000261180
Rattus norvegicus	ENSRNOP00055005256	NP_037179.1	EDL92741.1	N/A	ENSRNOP00065017403
Gallus gallus	ENSGALP00010032092	NP_990261.2	N/A	XP_004947106.2	XP_040515514.1
Anolis carolinensis	XP_003217792.2	XP_008106456.1	N/A	N/A	XP_062838686.1
Xenopus tropicalis	AAA49974.1	CAD12658.1	CAD12657.1	XP_002933212.1	XP_002939988.3
Danio rerio	ENSDARP00000021167	ENSDART00000181721.1 (TRH-R1a) ENSDART00000052509.7 (TRH-R1b)	ENSDART00000140654.2	XM_688966.3	ENSDARP00000083559
**Outgroup**					
Caenorhabditis elegans	C30H6.8	C30F12.6	N/A	N/A	N/A
Drosophila melanogaster	N/A	N/A	N/A	N/A	AAF56882.2

Biology Open • Supplementary information

Table S2. Primer sequences and qPCR validation parameters for target genes analyzed in this study.

Gene	Forward Primer (5′-3′)	Reverse Primer (5′-3′ antisense)	Amplicon Size (bp)	Dilution Range	Dilution Points	Slope	Efficiency (%)	R^2	Primer Source
trh	TGGAGCCGGAGGT GAGAA	CGAGTGGGGTCCTCTT ACGAT	91	$1:10^1–1:10^4$	4	-3.9842	78	0.99	Liang et al., 2015
trh-r1a	TTTTCCTACCACCA TCCCCG	TTGACCACCCATAACTG CCG	198	$1:10^1–1:10^6$	6	-3.8837	81	0.98	Designed in this study
trh-r1b	ACTGTGGAGCAGG GCCTCAT	ATGTCGAGCAGAACTG ACGA	295	$1:10^1–1:10^7$	7	-3.8223	82	0.98	Designed in this study
trh-r2	AAAGACCGGCTGA TGGGAGG	GAACTCCAGGTAGTGC GTGCT	179	$1:10^1–1:10^7$	7	-3.7311	85	0.99	Designed in this study
trh-r3	GTCTTCGCTCTGC TCTCGTT	CAGTCGGCAGGTCGCT TTGA	191	$1:10^1–1:10^4$	4	-3.8994	80	0.98	Designed in this study
trh-de	AGAGGTTCTCGGC TCGTGAC	CGAGTCTCGTGGTGTTT CGA	117	$1:10^1–1:10^7$	7	-3.8533	81	0.99	Designed in this study
insa	TAAGCACTAACCC AGGCACA	TCCTGGGCAGATTTAG GAGGA	151	$1:10^1–1:10^7$	7	-3.8523	81	0.99	Designed in this study
slc2a2	TTAACAGGCACGC TCGCTCT	TTCATGCTCTGTGCCAT TTCC	167	$1:10^1–1:10^5$	5	-3.1231	109	0.98	Designed in this study
slc2a12	GGGACAATCCTGG ACCACTA	ACATCCCAACCAGCATT CTC	156	$1:10^1–1:10^4$	4	-3.1889	105	0.98	Designed in this study
ins-ra	CGGTTCTGGGCAA AACAACA	GCAGGCCACGCATTTG TTAG	200	$1:10^1–1:10^5$	5	-3.9075	80	0.99	Designed in this study
actb1	TGAATCCCAAAGC CAACAGAG	CCAGAGTCCATCACAAT ACCAG	139	$1:10^1–1:10^6$	6	-3.1138	109	0.96	Designed in this study
18s rRNA	GAACGCCACTTGT CCCTCT	GTTGGTGGAGCGATTT GTCT	118	$1:10^1–1:10^5$	5	-3.3213	100	0.99	Designed in this study

All reactions were performed in a final volume of 8 µL and run in triplicate. Serial dilutions of cDNA were used to generate standard curves and determine amplification efficiency. All reactions were carried out using the same qPCR system described in the Methods section. No-template controls (NTCs) were included for each assay to rule out contamination or primer-dimer formation.

Biology Open • Supplementary information

Table S3. Primer sequences used for gene expression analysis

Primer	Oligonucleotide sequence			
	Forward (5′–3′)	Reverse (5′–3′)	Amplicon size (bp)	Accession number
trh	TGGAGCCGGAGGTGAAGA	GCAGTGGGGTCCTCTAGCAT	91	ENSDARG00000006868
trh-r1a	TTTTCCTCAACCCACTCCCG	TTGACCACCACTAACGTCCG	199	ENSDARG00000076546
trh-r1b	ACTGTCCTCTACGGCCTCAT	ATGTGCGACAGAACAGCAGA	295	ENSDARG00000036159
trh-r2	AAAGAACCGGCTGATGGAGG	GAATCCAGGTATGGCGTGCT	179	ENSDARG00000093901
trh-r3	GTCTTCGCTCTGCTCTGGTT	CAGTCGCAGAGCCTCTTGAA	191	XM_688966.3
trh-de	AGAAGGTTTCTGGCGGTGAC	CGAGCTCTCGTGTTTCAGA	117	ENSDARG00000109358
insa	TAAGCACTAACCCAGGCACA	TCCTGGGCAGATTTAGGAGGA	151	ENSDARG00000035350
slc2a2	TTAACAGGCACGCTCGCTCT	TTCATGCTCTGTGCCATTTCC	167	ENSDART00000078639.7
slc2a12	GGGACAATCCTGGACCACTA	ACATCCCAACCAGCATTCTC	156	ENSDARG00000036865
ins-ra	CGGTTCTGGGCAAAACAACA	GCAGGCCACGCATTTGTTAG	200	ENSDARG00000011948
ins-rb	CGGGGACGATATCCTGCAAA	ACGCATTTGGTGGAGTTCCC	187	ENSDARG00000071524
actb1	TGAATCCCAAAGCCAACAGAG	CCAGAGTCCATCACAATACCAG	139	ENSDARG00000113649
18s rRNA	GAACGCCACTTGTCCCTCT	GTTGGTGGAGCGATTTGTCT	118	XR_011015053
lsm12b	AGTTGTCCCAAGCCTATGCAATCAG	GACTCGTCTTTATCCTCCTGAGTGG	300	ENSDARG00000109510

Amplicon sizes (bp) were determined based on the expected PCR products using zebrafish transcript annotations. Gene abbreviations are as follows: *trh* (thyrotropin-releasing hormone), trh-r isoforms (*trh-r1a, trh-r1b, trh-r2, trh-r3*), and *trh-de* (thyrotropin-releasing hormone–degrading ectoenzyme). For glucose metabolism–related genes, *insa* corresponds to insulin isoform a; *slc2a2* and *slc2a12* encode the glucose transporters Glut2 and Glut12, respectively; and *ins-ra* and *ins-rb* represent insulin receptor isoforms a and b. Reference genes used for normalization included *actb1* (β-actin 1), *18S rRNA* (18S ribosomal RNA), and *lsm12b* (Like-Sm protein 12 homolog b). Accession numbers correspond to Danio rerio mRNA reference sequences retrieved from Ensembl and the NCBI GenBank RefSeq database.
